# Evolutionary history of barley cultivation in Europe revealed by genetic analysis of extant landraces

**DOI:** 10.1186/1471-2148-11-320

**Published:** 2011-11-02

**Authors:** Huw Jones, Peter Civáň, James Cockram, Fiona J Leigh, Lydia MJ Smith, Martin K Jones, Michael P Charles, José-Luis Molina-Cano, Wayne Powell, Glynis Jones, Terence A Brown

**Affiliations:** 1 NIAB, Huntingdon Road, Cambridge CB3 0LE, UK; 2 Manchester Interdisciplinary Biocentre, Faculty of Life Sciences, University of Manchester, Manchester M1 7DN, UK; 3 Department of Genetics, Faculty of Natural Sciences, Comenius University, Mlynská dolina B1, 842 15 Bratislava, Slovakia; 4 McDonald Institute for Archaeological Research, University of Cambridge, Downing Street, Cambridge CB2 3ER, UK; 5 Department of Archaeology, University of Sheffield, Northgate House, West Street, Sheffield S1 4ET, UK; 6 IRTA, Av. Alcalde Rovira Roure 191, 25198 Lleida, Spain; 7 Institute of Biological, Environmental and Rural Sciences, Aberystwyth University, Aberystwyth, Ceredigion SY23 3DA, UK

## Abstract

**Background:**

Understanding the evolution of cultivated barley is important for two reasons. First, the evolutionary relationships between different landraces might provide information on the spread and subsequent development of barley cultivation, including the adaptation of the crop to new environments and its response to human selection. Second, evolutionary information would enable landraces with similar traits but different genetic backgrounds to be identified, providing alternative strategies for the introduction of these traits into modern germplasm.

**Results:**

The evolutionary relationships between 651 barley landraces were inferred from the genotypes for 24 microsatellites. The landraces could be divided into nine populations, each with a different geographical distribution. Comparisons with ear row number, caryopsis structure, seasonal growth habit and flowering time revealed a degree of association between population structure and phenotype, and analysis of climate variables indicated that the landraces are adapted, at least to some extent, to their environment. Human selection and/or environmental adaptation may therefore have played a role in the origin and/or maintenance of one or more of the barley landrace populations. There was also evidence that at least some of the population structure derived from geographical partitioning set up during the initial spread of barley cultivation into Europe, or reflected the later introduction of novel varieties. In particular, three closely-related populations were made up almost entirely of plants with the daylength nonresponsive version of the photoperiod response gene *PPD-H1*, conferring adaptation to the long annual growth season of northern Europe. These three populations probably originated in the eastern Fertile Crescent and entered Europe after the initial spread of agriculture.

**Conclusions:**

The discovery of population structure, combined with knowledge of associated phenotypes and environmental adaptations, enables a rational approach to identification of landraces that might be used as sources of germplasm for breeding programs. The population structure also enables hypotheses concerning the prehistoric spread and development of agriculture to be addressed.

## Background

Cultivated barley (*Hordeum vulgare *L.), the domesticated form of *Hordeum spontaneum *C. Koch, was one of the founder crops of agriculture in western Asia, first appearing in the archaeological record in the 8^th ^and 7^th ^millennia BC [1,2]. Barley was also one of the principal crops that accompanied the spread of agriculture into Europe during the 6^th ^and 5^th ^millennia BC. Today it is grown throughout the continent, mainly for animal feed and malt for brewing [3].

Until the introduction of modern cultivars in the mid-20^th ^century, European barley comprised a large number of landraces, each of these a 'dynamic population or populations of a cultivated plant that has historical origin, distinct identity and lacks formal crop improvement, as well as often being genetically diverse, locally adapted and associated with traditional farming systems' [4]. Many landraces died out during the last century, but seeds representing several thousand types from all parts of Europe are available from germplasm collections [5]. Increasingly, these landrace collections are being looked on as important sources of germplasm with which to enrich the genepool of modern barley cultivars [6]. Exploitation of these landraces in modern crop breeding requires understanding not only of their phenotypic attributes and environmental adaptations, but also their evolutionary relationships. This information would enable landraces with similar valuable traits but different genetic backgrounds to be identified, providing alternative strategies for the introduction of the traits into modern germplasm.

An understanding of the relationships between different landraces might also provide information relating to the spread and subsequent development of barley cultivation in Europe. This possibility has been recognized for some time [7], but has not been extensively explored. Recent studies have suggested, however, that European barley landraces are differentiated into genetically defined populations. For example, DNA sequencing of the photoperiod response gene *PPD-H1 *in European barley landraces has revealed three distinct groups of haplotypes, two (groups B and C) with the daylight responsive phenotype found in most wild barleys, and one (group A) with the derived nonresponsive phenotype that confers adaptation to the long annual growth season of northern Europe [8]. Multilocus studies have also been carried out, avoiding the risks of inferring population history from single gene data. Population structure, linked at least in part with ecogeography and/or agronomic factors, has been revealed by analysis of microsatellites in barley landraces from Iberia [9], the Levant [10] and the Himalayas [11]. A relationship between microsatellite genotypes and ecogeography has also been demonstrated at the microscale for wild barley [12].

In this paper we show that European barley landraces can be divided into populations based on their microsatellite genotypes. We assess the extent to which the population structure can be explained by human selection, environmental adaptation, geographical partitioning occurring during the initial spread of barley cultivation into Europe, and/or the later introduction of daylength-nonresponsive landraces.

## Results

### Microsatellite genotypes

We studied 651 accessions of cultivated barley (Additional file 1, Table S1) and typed 24 microsatellites (Additional file 1, Table S2). Each of these microsatellites displayed variability among the landraces that were tested, the number of alleles observed per locus ranging between 2-26 with a mean of 9.0 (Additional file 1, Table S3). PIC values varied between 0.05-0.90 (mean 0.49), in broad agreement with values previously obtained for Spanish barley landraces [9]. There were no significant differences between the overall diversities of two- and six-rowed barley landraces, though several individual microsatellites did show significant differences when these two sets of landraces were compared. Missing data (i.e. landraces that gave no PCR product for a particular microsatellite) varied from 0.8-41.9% (mean 9.6%). Missing data, which usually arise when a landrace has a sequence polymorphism within the annealing site for one of the two primers used to amplify the microsatellite, will lead to underestimates of overall diversity, but are not an issue for subsequent data analysis because STRUCTURE is able to compensate for gaps in the overall dataset.

### Identification of populations

To assess whether the barley landraces could be divided into populations, STRUCTURE was used to calculate the probability distribution Pr(*X*|*K*) for values of *K *from 2-20, where *X *is the genotypes of the sampled landraces and *K *is the number of populations. Multiple runs were carried out and population assignments tested for reproducibility by mutual correlation of the Q-matrix outputs [13,14]. Q-matrices were accepted as reproducible if the allocations to each population could be identified as similar in a pair of Q-matrices and were highly correlated (r > 0.999). For *K *= 2, 3, 5, 9, 10, 14 and 15 reproducibility was achieved after duplicate runs, while for *K *= 4, 6, 7, 8, 11, 12 and 13 two reproducible results were obtained after a third run. The population assignments at *K *= 16 were symmetrical, each accession being given an equal allocation (0.0625) to every population, and reproducible results could not be obtained for values of *K *from 17-20. These results indicate that the dataset displays population structure and that the most likely value of *K *is ≤15.

**Figure 1:**
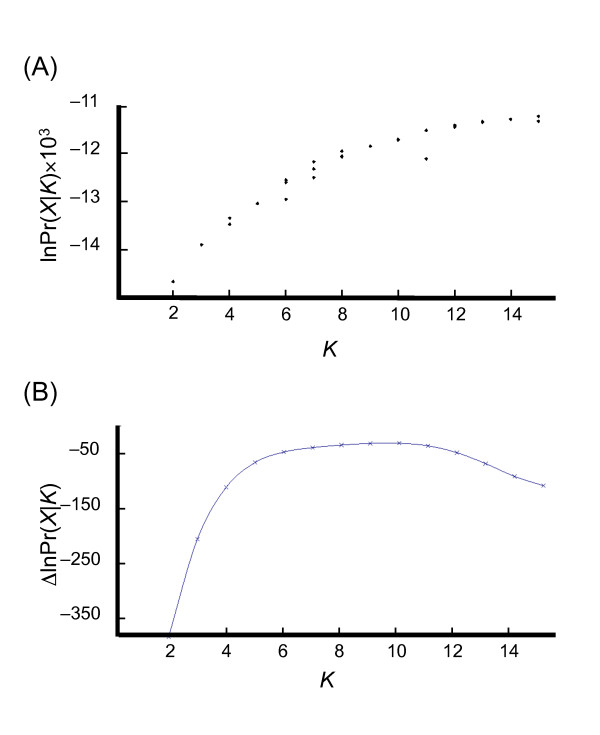
**Identification of the most likely value for *K***. (A) Identification of the smallest value of *K *that captures the major structure in the microsatellite data. The graph shows the increase of lnPr(*X*|*K*) against *K *for *K *= 2 to 15. (B) Estimation of the most likely value of *K *from the position of the plateau in a plot of the rate of change of lnPr(*X*|*K*), estimated from (a), against *K*.

**Figure 2:**
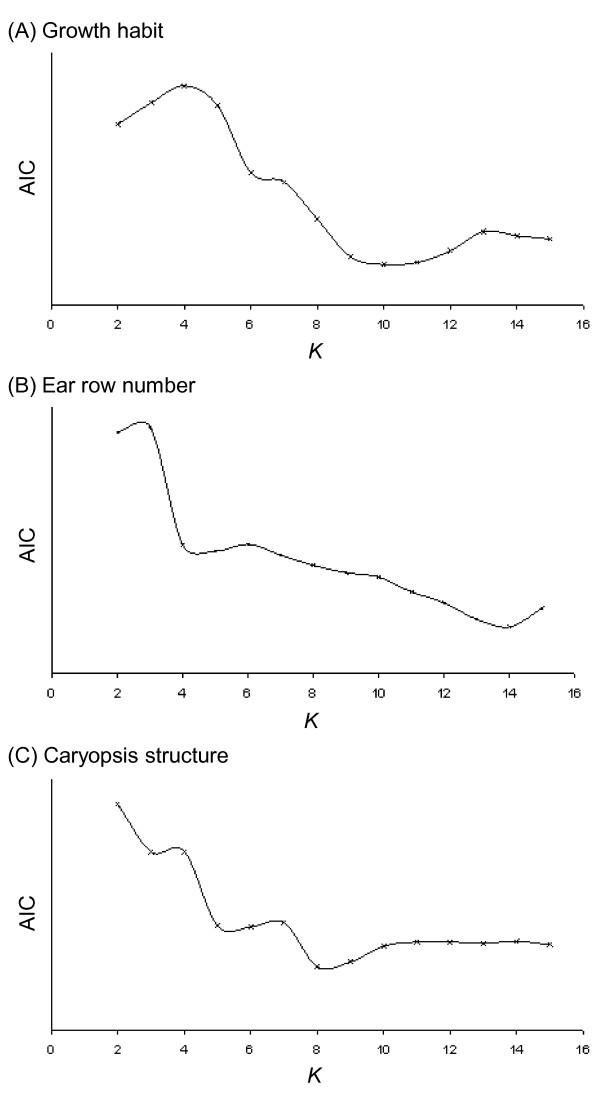
**Using the expected relationship between genetic structure and phenotype to identify likely values for *K***. (A) Spring or winter growth habit; (B) 2-row or 6-row ear morphology; (C) hulled or naked caryopsis.

Three methods were used to identify the most likely value of *K *more accurately. The first method was based on the prediction that the upper limit of *K *is indicated by the smallest value that captures the major structure in the data [15]. A plot of lnPr(*X*|*K*) against *K *was therefore made for *K *= 2 to 15 (Figure 1A). This plot shows lnPr(*X*|*K*) increasing until *K *= 12-13, after which the line reaches a plateau. This observation suggests that the most likely value of *K *is ≤12. In the second method the rate of change of lnPr(*X*|*K*) for successive values of *K *was plotted against *K *(Figure 1B). The position of a plateau in a plot of this type indicates the most likely value(s) of *K *[16]. Examination of Figure 1B therefore suggests that *K *= 9-10. The third method used to identify the most likely number of populations assumes that genetic structure should relate to phenotype [17]. Data on spring or winter growth habit, two-row or six-row ear morphology and caryopsis structure were converted into binary characters and logistic regressions of these phenotypes against the Q-matrices for *K *= 2-15 carried out with the R statistics package. In this type of analysis, the lowest values for Akaike's information criterion **(**AIC) identify the range of *K *where population structure is best correlated with phenotype. For growth habit, the lowest AIC values were found for *K *= 9-11, for row number at *K *= 14, and for caryopsis structure at *K *= 8-9 (Figure 2). Taken together, the three analyses summarized in Figures 1, 2 suggest that the most likely value of *K *for the barley landraces is between 8 and 11. While we do not attempt to assign a 'correct' value for *K*, we chose a value of 9 as a starting point for examination of population structure.

**Figure 3:**
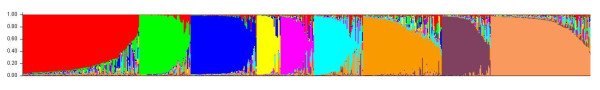
**Graphical representation of population structure for barley landraces at *K *= 9**. Each landrace is shown as thin vertical segment whose colour(s) indicates its proportional membership(s) of each population.

A graphical representation of the population structure at *K *= 9 revealed that each population was partially admixed with other populations, the overall degree of admixture being similar for each one (Figure 3). Half the landraces (335/651) had a proportional membership of ≥0.9 for their primary population, and only 51 had a primary proportional membership of < 0.5.

**Figure 4:**
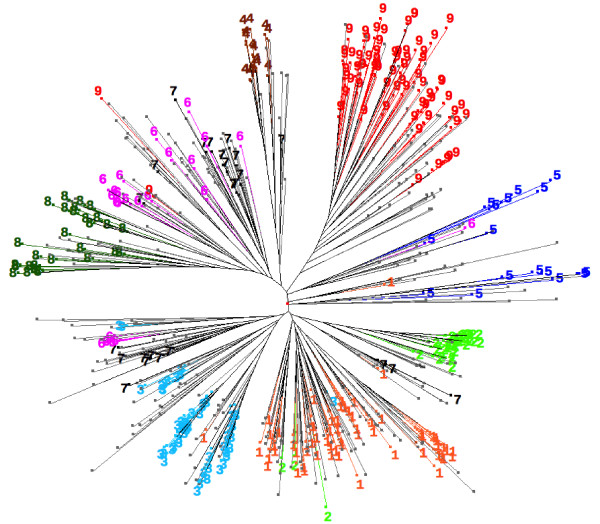
**Neighbour-joining tree constructed from the microsatellite genotypes of all accessions**. The positions of those accessions with a proportional membership of ≥0.9 in their primary population are marked.

The relationships between these populations were studied in two ways. First, the microsatellite data were used to construct a neighbour-joining tree and those accessions with a proportional membership of ≥0.9 in their primary population marked on the tree (Figure 4). Accessions belonging to a single population clustered together, except for population 7, and to a lesser extent 6, which appeared in two distinct regions of the tree. Populations 1-3 and some members of 6 and 7 grouped close to one another, as did population 8 alongside the remainder of 6 and 7. Populations 4, 5 and 9 each formed a separate group, although population 5 was split into two parts each with its own deep root towards the base of the tree.

**Figure 5:**
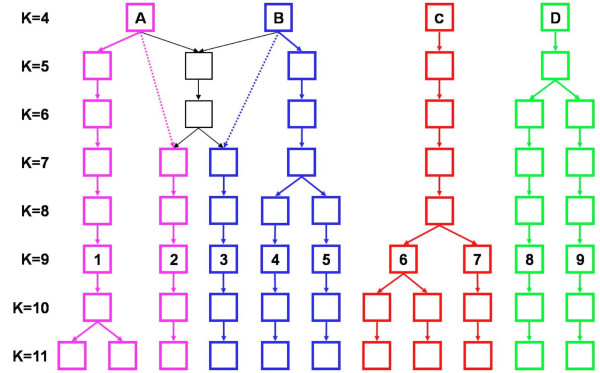
**Hierarchical pattern of population structure at values of *K *from 4 to 11**.

To explore further the population relationships, the groups resulting from STRUCTURE analyses with preset *K *values of 4-11 were investigated. Again, only those landraces that displayed a proportional membership of ≥0.9 in their primary populations were considered. Comparing the allocations for each of these landraces as *K *increased revealed a hierarchical structure to the landrace populations (Figure 5). Landraces that were grouped together at *K *= 4 were still grouped together at *K *= 7-11. A subset of landraces from two of the populations at *K *= 4, A and B, were placed in a shared population at *K *= 5-6, then at higher *K *values re-sorted into distinct lineages that corresponded to their original allocation between populations A and B. These landraces made up populations 1-5 at *K *= 9. The remaining two populations at *K *= 4, C and D, both gave rise to lineages that remained distinct as *K *increased to 11.

### Phenotypic features of the populations

For those accessions for which data were available, ear row number, caryopsis structure, growth habit and daylength responsiveness were compared with the population structure (Table 1). Two-rowed barley predominated in populations 1-3 (two-row > 90%) and six-row barley predominated in populations 5-9 (six-row > 60%), these differences being significant (χ^2 ^> χ^2^_critical_, p < 0.05). Naked barleys were predominant in population 4 (naked 75%), and formed a sizeable minority of population 5 (naked 28%), but were virtually absent from the other seven populations (naked 0-3%). Winter growth habit was predominant only in population 8 (65% winter according to the passport data, 60% winter according to *VRN-H1 *and *VRN-H2 *typing). Spring growth habit accounted for > 90% of the accessions in populations 1-4 and 7 according to passport data, and in population 6 also according to *VRN *genotype. The remaining two populations (5 and 9) comprised a mixture of spring and winter barleys. The discrepancies within populations 6 and 9 between the passport data for growth habit and the habit predicted from the *VRN *genotype are due to many of these accessions coming from the Mediterranean region, where barley with a facultative growth habit is favoured. These barleys are autumn sown, able to survive mild winter conditions, and flower in the late spring. They are therefore recorded as having a winter growth habit although they display the spring genotype when the *VRN *genes are typed.

**Table 1:** Phenotype data for the barley landraces included in this study

Population	Number of landraces	Ear row number	Caryopsis structure	Growth habit - morphology	Growth habit - *VRN *genotype	Daylight responsiveness
		**(2 row:** **6 row)**	**(hulled:** **naked)**	**(spring:** **winter)**	**(spring:** **winter)**	**(responsive:** **nonresponsive)**

1	135	129: 2 (131) 98:2	129: 4 (133) 97:3	126: 1 (127) 99:1	30: 1 (31) 97:3	0: 32 (32) 0:100

2	60	59: 1 (60) 98:2	60: 0 (60) 100:0	56: 4 (60) 93:7	7: 0 (7) 100:0	2: 35 (37) 5:95

3	77	72: 5 (77) 94:6	75: 2 (77) 97:3	76: 0 (76) 100:0	2: 0 (2) 100:0	0: 30 (30) 0:100

4	28	20: 8 (28) 71:29	7: 21 (28) 25:75	28: 0 (28) 100:0	2: 0 (2) 100:0	0: 12 (12) 0:100

5	36	13: 23 (36) 36:64	26: 10 (36) 72:28	22: 10 (32) 69:31	6: 3 (9) 67:33	3: 6 (9) 33:67

6	57	7: 49 (56) 13:87	56: 0 (56) 100:0	36: 19 (55) 65:35	23: 2 (25) 92:8	16: 9 (25) 64:36

7	92	19: 67 (86) 22:78	91: 0 (91) 100:0	84: 7 (91) 92:8	35: 2 (37) 95:5	15: 23 (38) 39:61

8	57	8: 49 (57) 14:86	57: 0 (57) 100:0	18: 33 (51) 35:65	3: 6 (9) 33:67	22: 0 (22) 100:0

9	109	7: 102 (109) 6:94	108: 1 (109) 99:1	71: 34 (105) 68:32	22: 4 (26) 84:16	23: 2 (25) 92:8

^a ^Top row, number of landraces with each phenotype, with the total that could be phenotyped in brackets; bottom row, percentage of landraces with each phenotype

For 240 accessions, daylength responsiveness was deduced from the *PPD-HI *genotype [8] (Additional file 1, Table S4). All of the accessions of populations 1, 2 and 4 that were typed, as well as 95% of those in population 3, were daylength nonresponsive, and all those typed from population 8 and 91% of those from population 9 were responsive. The other four populations were mixed, ranging from 20-91% responsive. Where possible, those accessions with the daylength responsive genotype were placed in group B or C [8]. Group B predominated (> 70%) only in population 9 and ranged from 0-13% in the other populations.

### Geographical distributions of the populations

**Figure 6:**
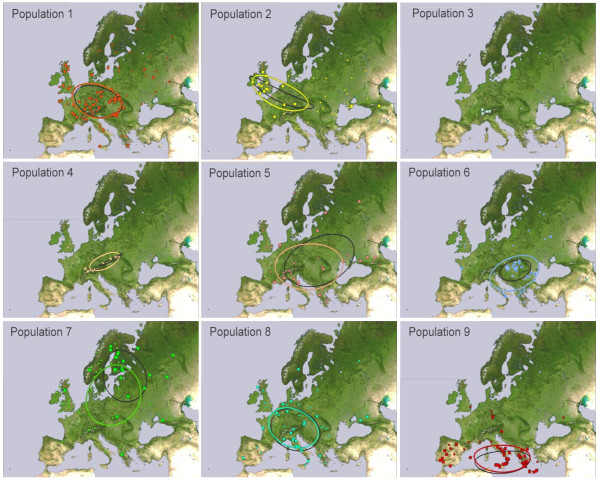
**Geographical distributions of landraces for each of the barley populations identified at *K *= 9**. The locations of the individual landraces are indicated and the circles are the standard deviation ellipses for each population. The locations of those landraces with a proportional population membership of ≥0.9 are shown by the large squares and their standard deviation ellipses are drawn in black. Landraces with a proportional population membership of < 0.9 are shown by the small dots, and the standard deviation ellipses obtained when these landraces are included in the analysis are drawn in colour. For an expanded view of the population 3 distribution, see Additional file 1, Figure S1.

All 651 accessions were included in the geographical analyses. The mean centre and standard distance for each population (equivalents to the mean and standard deviation of a numeric distribution) were calculated, along with the mean pairwise geographical distance within each population to assess the degree of clustering (Additional file 1, Table S5). All of the landraces were included. Landraces of each population were placed on a map of Europe and standard deviation ellipses plotted (Figure 6). Visual inspection of the maps indicated that the populations had non-identical distribution patterns. Three populations (1, 5 and 8) were distributed over a relatively broad area of central and western Europe. Population 2 had a similar but more western distribution, largely due to a preponderance of British landraces. Populations 3 and 4 centred on Switzerland and the Carpathian mountains, with population 3 tightly clustered in a small area of west-central Switzerland. Population 6 was mainly located in the Balkan region of southeast Europe, population 7 predominated in north central Europe including Scandinavia and the Baltic States, and population 9 clustered in the Mediterranean region.

To assess the apparent differences between these distribution patterns, the great circle distances between pairs of populations were tested for significance using Student's t-test (p < 0.05 or p < 0.01, 1-tailed) against a null hypothesis that the mean centres were 0 km apart. This analysis suggested that 28 of the 36 possible population pairs had a highly significant (p < 0.01) difference in their mean centres, and a further four (1 and 3, 1 and 4, 4 and 8, 5 and 8) had a significant (p < 0.05) difference. The only non-significant differences were between paired populations 1 and 8, 3 and 8, 4 and 5, and 5 and 6.

The relationship between the population structure and environment was investigated by comparing the between-population variance and within-population variance for a range of climatic factors. This analysis was carried out separately for the spring and winter accessions (based on passport data) as these different growth habits might be expected to result in different patterns of environmental adaptation. For randomized populations, the F ratio for between-population variance compared with within-population variance was close to one for each of the climate variables at all months of the year. When all the climate variables were combined, the between-population variance for spring barleys was significantly higher than within-population variance for each month, with the highest values occurring during the growing season for these accessions (Table 2). Between-population variance was also significantly greater than within-population variance for winter barleys when all climate variables were combined (Table 2) but with no obvious seasonal trend.

**Table 2:** Data for between-population variance: within-population variance for a series of climate variables

Month	Spring barleys		Winter barleys	
	**F**	**-log P**	**F**	**-log P**

January	38.9	48	4.3	4

February	69.1	76	6.5	6

March	87.7	90	7.2	7

April	107.4	103	5.1	5

May	153.0	129	5.3	5

June	158.1	132	5.8	5

July	189.1	146	9.0	8

August	187.0	145	9.7	9

September	162.1	134	8.7	8

October	111.6	106	5.0	5

November	76.3	81	4.5	4

December	74.6	80	5.4	5

## Discussion

### Identification of populations

STRUCTURE was used to determine whether European barley landraces can be divided into populations based on their microsatellite genotypes. In this context, a population is defined as a group of individuals that share a characteristic set of allele frequencies at the loci that are studied [15]. STRUCTURE places individuals in populations in such a way as to minimize within-population deviations from Hardy-Weinberg equilibrium and linkage equilibrium. It therefore assumes that the individuals are fully outcrossing, and modelling studies have suggested that with partially inbreeding taxa the results of STRUCTURE analysis can be spurious [18,19]. Although cultivated barley has an outcrossing rate of less than 2% [20], the outcomes of previous STRUCTURE analyses of genetic data from barley have been supported by other analyses of the same data and are in agreement with the conclusions of earlier and later work [e.g. [21,22]]. In these projects it therefore appears that STRUCTURE identified authentic populations despite barley's low outcrossing rate. An explanation might be provided by other simulations which have shown that, over hundreds of generations, the pattern of multilocus marker inheritance in a population of plants displaying 2% outbreeding is indistinguishable from that displayed by a panmictic population [23].

The results of our STRUCTURE analysis were reproducible for values of *K *up to 15 and, using standard methods, we concluded that the most likely number of populations was between 8-11. The population structure was hierarchical, and from *K *= 7-11 the only effect of each incremental increase in *K *was to subdivide an existing population with the memberships of the other populations remaining unchanged (Figure 5). This consistent pattern of population assignment indicates that the results of the STRUCTURE analysis were not spurious. To further assess the validity of the analysis, we constructed a neighbour-joining tree for all 651 accessions and marked the positions of those accessions with a proportional membership of ≥0.9 in their primary population at *K *= 9 (Figure 4). Accessions belonging to a single population clustered together, with the exception of populations 6 and 7, whose members are distributed in two parts of the tree. The tree topology therefore provides independent support for the STRUCTURE results, and also suggests that it is reasonable to use *K *= 9 as the basis for interpretation of the population structure.

### Association between phenotype and population structure

For a domesticated plant such as cultivated barley, one possible way in which population structure could arise is as a result of selection for particular phenotypic traits. The phenotypes of greatest agronomic importance in modern farming are ear row number, caryopsis structure (hulled or naked grains), growth habit and flowering time. Wild barley has a two-rowed ear, each spikelet having a fertile central floret flanked by two infertile laterals which, when combined with a long awn, takes on an arrowlike form that is an effective aid to seed dispersal and burial [24]. Many cultivated barleys retain this ancestral head structure but in the derived six-rowed form the two lateral florets are fertile. Six-rowed barley is more often used as an animal or human feed, whereas two-rowed barley is favoured for malting and brewing. Wild and most cultivated barleys have hulled grains where the outer lemma and inner palea adhere to the pericarp epidermis at maturity. This form is favoured by brewers because the hull debris aids wort filtration, whereas the free-threshing 'naked' varieties are preferred when barley is grown for direct human consumption [25]. Wild barley has a winter growth habit, meaning that it requires vernalization - exposure to a prolonged period of cold - in order to promote subsequent flowering. The majority of European landraces lack this requirement and have a spring growth habit, where plants avoid periods of cold weather by completing their growth cycle during a single season, rather than over wintering as plants in a vegetative state [26]. Finally, most wild barleys display a photoperiod response that triggers flowering early in the season, before the conditions become too dry for further vegetative growth. Many cultivated barleys, especially landraces from northern Europe, are daylength nonresponsive, and so continue vegetative growth until flowering later in the summer [8], allowing them to take advantage of the longer growing season in northern Europe.

We compared phenotypic data for ear row number, caryopsis structure, growth habit and flowering time with the population memberships (Table 1). Populations 1-3 display a similar set of phenotypic features, most of these accessions being two-rowed (99, 98, 94% for populations 1-3, respectively), hulled (98, 100, 97%), spring habit (99, 93, 100%), and daylength nonresponsive (100, 95, 100%). Populations 6 and 9, which contained a high proportion of accessions with the facultative growth habit, also show some similarities when other phenotypes are considered, being largely six-row (87% for population 6, 94% for population 9), hulled (100, 99%) and daylength responsive (67, 91%). In population 6, however, the majority of the responsive accessions were members of group C (81% of responsive accessions), whereas in population 9 the majority were group B (80%), these two groups having distinct evolutionary histories [8]. No other similarities between the range of phenotypes displayed by different populations were apparent. These comparisons indicate that there is a degree of association between phenotype and population structure, suggesting that selection may have played a role in the origin and/or maintenance of one or more populations.

### Association between climatic factors and population structure

The spread of agriculture involved the dispersal of barley well beyond the native range of the wild species into the variety of environments found in Europe. Adaptation to these new conditions is reflected in a north-south clinal distribution of landraces with the daylength responsive and nonresponsive genotypes of the photoperiod gene *PPD-H1*, nonresponsive forms more common in the cooler northern latitudes [8]. With wild barley, there is a strong correlation between population structure and temperature and precipitation [27]. It might therefore be anticipated that similar climatic correlations may be discernable in the population structure of cultivated barley.

Analysis of a series of climate variables supported these expectations (Table 2). Between-population variance was significantly higher than within-population variance for both spring and winter barleys. This trend was apparent at all months of the year, but for spring barleys was strongest during the growing season. For winter barleys the seasonal trend was less clear. The results indicate that the accessions in each population are adapted, at least to some extent, to their environment, but do not reveal whether this adaptation was a factor in the origin of individual populations, or merely reflects the more recent evolution of landraces to the environments in which they are being grown.

### Origins of the populations

The relationships inferred from the groupings revealed by neighbour-joining (Figure 4), along with the phenotypic and geographic data, enable possible origins for the populations to be deduced.

Populations 1-3 are closely related, forming a distinct group in the neighbour-joining tree, and have identical phenotypes, virtually all of their members being two-rowed and hulled with spring growth habit and daylength nonresponsiveness (Table 1). We have previously shown that the nonresponsive phenotype of European barleys originated in the eastern Fertile Crescent and that the first nonresponsive plants probably entered Europe after the initial spread of agriculture [8]. This population of nonresponsive plants would almost certainly have had a distinct genetic makeup compared with the barley already present in Europe, which originated in the western Fertile Crescent. Populations 1-3 are almost exclusively nonresponsive (of the 99 accessions from these three populations that were typed, 97 possessed a nonresponsive haplotype) and could be the descendents of this original population of nonresponsive plants. These three populations possess the wild phenotypes for ear row number and caryopsis structure, but have acquired a spring growth habit, whereas their wild progenitors would have been winter types. The presence of some members of population 7 in the same region of the neighbour-joining tree as populations 1-3 is indicative of past cross-hybridisation between these populations, which we discuss below.

Population 4 is also made up entirely of daylength nonresponsive accessions. This population is located some distance from populations 1-3 in the tree topology. Population 4 has a narrow geographical distribution in Switzerland and the Carpathian mountains (Figure 6) and is the only population in which the majority of accessions have naked rather than hulled grains. The apparent lack of a close relationship between population 4 and populations 1-3 might indicate that the former is not directly descended from the latter. Instead, population 4 could have become homogeneous for daylength nonresponsiveness via a founder effect operating on a population that contained a mixture of responsive and nonresponsive types. The tree topology suggests that this progenitor of population 4 might have been related to the modern populations 6 and/or 7.

Population 5 forms a separate cluster in the neighbour-joining tree, but has a mixture of phenotypes, including two- and six-row barleys, hulled and naked forms, spring and winter habits and both daylight responsive and nonresponsive. There is little uniformity to the combination of phenotypes possessed by individual accessions, and the two deeply rooted groups within the population 5 cluster are equally mixed. These features, along with the broad geographical distribution, suggests that this population has not been subject to selection. With a crop such as barley, one way in which a distinct genetic population might arise is by geographical partitioning during or soon after the initial spread of agriculture. Populations might be expected to arise in this way if the process of spread involves two or more trajectories that isolate different parts of the crop so that cross-hybridization between the nascent populations is restricted. The original spread of agriculture into Europe is thought to have followed at least two trajectories, one along a northern route through the Balkans, Hungary and Danube and Rhine valleys, and the other through the Mediterranean basin to Italy and Iberia [28-30]. The lack of evidence for human or environmental selection might therefore indicate that population 5 is a relict of a population that originated from the geographical partitioning that occurred during this initial period of spread along the northern trajectory.

Another candidate as a relict is population 9, as the core area of distribution of this population lies within those regions of Mediterranean Europe where crops are thought to have spread via the southern trajectory. If the spread of cultivation along this trajectory resulted in evolution of a distinct population of barley then that population, at least initially, would have had a geographical distribution very similar to that displayed today by population 9.

Population 9 is predominantly six-rowed, hulled and daylight responsive, with a mixture of winter and spring types. Population 8 has similar phenotypic features to population 9 but contains a greater proportion of landraces with the winter growth habit and is exclusively daylight responsive, whereas population 9 includes some nonresponsive types. The possibility that the two populations might have an evolutionary relationship is supported by the STRUCTURE analysis, the two populations being grouped as one at *K *= 4, not splitting into separate populations until *K *= 6 (Figure 5), but the topology of the neighbour-joining tree gives less evidence for a close relationship.

The final two populations, 6 and 7, are grouped as one by STRUCTURE at *K *≤ 8, and their accessions are located together in the neighbour-joining tree, albeit in three separate parts of the topology. Their geographical distributions are largely non-overlapping, with population 6 centering on the northern Balkans, Hungary and Romania, and population 7 in northern Europe, Scandinavia and the Baltic States. This suggests that originally they formed a single population spanning most of the eastern half of Europe, subsequently splitting into two, possibly by geographical partitioning. They are largely six-row, entirely hulled and predominantly spring growth habit, but they contain a mixture of daylength responsive and nonresponsive forms. The latter are located almost exclusively within the lower part of the tree shown in Figure 4, alongside populations 1-3. The implication is that cross-hybridization resulted in transfer of the daylength nonresponsive phenotype from populations 1-3 to some members of populations 6 and 7. Daylight nonresponsiveness and spring growth habit can be advantageous for the successful growth of barley in the more northerly regions of Europe. Acquisition of daylength nonresponsiveness by a group of early barley landraces that had already evolved a spring growth habit might therefore have been one of the evolutionary adaptations that enabled cultivation of those plants to be extended further north into the regions now occupied by populations 6 and 7. It might therefore be hypothesized that these populations represent a derived form of barley that evolved during the spread of agriculture into central and northern Europe. We explore these and other archaeological interpretations of the population structure in more detail elsewhere (Jones et al., in preparation).

## Conclusions

We have shown that barley landraces can be divided into populations based on their microsatellite genotypes, and that these populations have different core distributions in Europe. The population structure is partly associated with phenotype, suggesting that human selection and/or environmental adaptation may have played a role in the origin and/or maintenance of one or more populations, but there is also evidence that at least some of the population structure originated during the initial spread of barley cultivation into Europe, or reflects the later introduction of daylength-nonresponsive varieties. The dissection of population structure, combined with examination of their phenotypic attributes and environmental adaptations, enables a rational approach to the identification of landraces that might be used as sources of valuable germplasm for modern breeding programmes.

## Methods

### Plant material and phenotype data

The 651 accessions of cultivated barley included in this study are listed in Additional file 1, Table S1. All were described by the germplasm suppliers as landraces or traditional cultivars. The accessions were chosen to give full geographical coverage across Europe. Information on seasonal growth habit (winter or spring), ear row number and caryopsis structure (hulled or naked grains) were obtained from the passport data for each accession. If not given in the passport data, ear row number and caryopsis structure were identified from the grain morphology. For 149 of the accessions, growth habit was also predicted from the genotypes of the vernalization loci *VRN-H1 *and *VRN-H2 *[26,31]. For 148 accessions, daylength responsiveness was inferred from the genotype of the photoperiod response gene *PPD-H1 *using our published data [8], and for another 82 accessions by typing the causative SNP within *PPD-H1 *(Additional file 1, Table S4).

### Microsatellite genotyping

In order to analyse population structure, a single genotype must be assigned to each accession. Some barley landraces are genetically diverse, and it cannot be assumed that the genotype of a single plant taken at random from the accession will be representative of the landrace as a whole. To avoid such errors, microsatellite genotypes were determined for two bulk samples per accession, each sample composed of a different set of ten coleoptiles, the original seeds chosen at random, and the most frequent allele identified in those cases where a landrace gave a mixed genotype. DNA was prepared using the Qiagen DNeasy96 kit and PCRs directed at 24 microsatellite loci using the primer pairs listed in Additional file 1, Table S2, set up as 10 μl reactions containing 1 μl of DNA extract, 1 × PCR buffer with MgCl_2 _(Roche), 0.2 mM each dNTP, 0.5 μM primers and 0.1 units Taq DNA polymerase (Roche). PCRs were performed using a GeneAmp PCR System 9700 (Applied Biosystems) as multiplexes of up to four primer pairs per reaction, using the following cycling conditions: 94°C for 1 min; 7 cycles of 94°C for 50 seconds, 65°C for 30 seconds decreasing by 1°C per cycle, 72°C for 30 seconds; 28 cycles of 94°C for 50 seconds, 58°C for 30 seconds, 72°C for 30 seconds; and a final extension of 72°C for 5 min. PCR products were analysed in a PRISM 3100 Genetic Analyzer (Applied Biosystems). Data were recorded and microsatellite allele lengths measured using the Genemapper 3.7 software (Applied Biosystems). For SSR 12, two sets of alleles were recorded independently as SSR 12A (which included the range of alleles observed in elite barley cultivars) and SSR 12B (which included alleles of greater length). In those cases where a DNA extract gave peaks for multiple alleles, the amplicon giving the most intense signal was recorded. The use of duplicate assays allowed an internal check for data quality, reducing the likelihood of a minority allele mistakenly being recorded. This approach is more straightforward than more complex methods for assigning allele frequencies in mixed microsatellite genotypes, such as thresholding [32] and calibration [33], and is equally accurate when only the most frequent allele is being recorded.

For each microsatellite, summary data including the number of alleles observed, major allele frequencies, gene diversities and polymorphism information contents (PIC), were calculated using Powermarker version 3.25 [34]. The R statistics package [35] was used to calculate genetic distance between accessions and the APE and ADEGENET programs used to construct neighbour-joining trees depicting these relationships.

### Analysis of population structure

Population structure was evaluated using STRUCTURE 2.2 [15] with a burn-in of 200, 000 followed by 1, 500, 000 Markov Chain Monte Carlo iterations. The haploid setting and admixture model for ancestry between individuals were chosen, a degree of admixture being a reasonable expectation for populations of landraces that have had opportunities for cross-pollination. Statistical analysis of data generated by STRUCTURE was performed using the R statistics package.

ArcGIS 9.0 (ESRI) was used to analyse the geographical distributions of accessions belonging to different populations. Those accessions supplied without detailed information on sampling location were assigned latitude and longitude representing the country of origin (National Geospatial Intelligence Agency 'Country Coordinates': http://earth-info.nga.mil/gns/html/gis_countryfiles.htm). Correlations between the point of origin for each accession and climatic data were examined by analysis of variance. The climatic data were month-by-month averages for the period 1921-1940 for near-surface mean, minimum and maximum temperatures, diurnal temperature range, precipitation, wet day frequency, frost day, vapour pressure and cloud cover, collated from the CRU TS 2.1 Global Climate Database [36]. The data were summarized by calculating an overall mean for each accession, monthly means for all climate variables and variable means for all months. A logistic regression between the climate variables and the proportional membership of each accession in its population was performed, and the partition of variation examined in an analysis of variance. Actual climate data from 1921-1940 was used rather than inferred data for earlier periods. Although there have been short-term variations over the 8500 years since barley was introduced into Europe, the geographical variations existing in the past are likely to be reflected in the geographical variations in the recorded measurements for 1921-1940. The advantage of the recorded measurements is that they enable much greater precision in identification of the specific data pertaining to the collection points for individual landraces.

## Authors' contributions

HJ conducted most of the research and data analysis. PC carried out additional typing of the *PPD-HI *locus. HW, TAB, GJ and JC carried out the data analysis. HJ, FJL, LMJS, MKJ, MPC, WP, GJ and TAB jointly conceived and supervised the project as part of the Domestication of Europe consortium. JLMC provided material from Spain as part of a collaborative arrangement with the consortium. HJ, GJ and TAB drafted the manuscript. All authors drafted and approved the final manuscript.

## Supplementary Material

Additional file 1 **Additional file for 'Evolutionary history of barley cultivation in Europe revealed by genetic analysis of extant landraces'**. Contains Table S1 Barley accessions used in this study, Table S2 Microsatellite loci and PCR details, Table S3 Microsatellite data for the 651 barley landraces, Table S4 *PPD-H1 *genotypes for 82 barley landraces, Table S5 Geographical data, Figure S1 Expanded view of the core distribution of population 3. Click here for file
