# Supplementary material for: Evolutionary history of barley cultivation in Europe revealed by genetic analysis of extant landraces
Source: BMC Evol Biol. 2011 Nov 2;11:320. doi: 10.1186/1471-2148-11-320 (PMC3248229; doi:10.1186/1471-2148-11-320)

**Evolutionary history of barley cultivation in Europe revealed by genetic analysis of extant landraces**

Huw Jones, Peter Civáň, James Cockram, Fiona J. Leigh, Lydia M.J. Smith, Martin K. Jones, Michael P. Charles, José-Luis Molina-Cano, Wayne Powell, Glynis Jones, Terence A. Brown

**ADDITIONAL FILE**

Table S1 lists the barley accessions used in this study. ‘Source number’ is the reference number used by the germplasm collection that supplied the accession. ‘Collection site’ is as described in the passport data except for ‘traditional varieties’, for which the germplasm collection’s accession name and description is used. ‘Country codes’ are abbreviations as follows: AFG, Afghanistan; ALB, Albania; AUS, Australia; AUT, Austria; BEL, Belgium; BGR, Bulgaria; BOS, Bosnia and Herzogovina; BYS, Byelorus; CHE, Switzerland; CZE, Czech Republic; DEU, Germany; DNK, Denmark; ESP, Spain; EST, Estonia; FIN, Finland; FRA, France; FRO, Faroe Islands; GBR, Great Britain; GEO, Georgia; GRC, Greece; HRV, Croatia; MAG, Hungary; IRL, Eire; ISR, Israel; ITA, Italy; LAT, Latvia; LBY, Libya; LIT, Lithuania; MAR, Morocco; NLD, Netherlands; NOR, Norway; POL, Poland; ROM, Romania; RUS, Russia; SLV, Slovenia; SUN, Union of Soviet Socialist Republics; SVK, Slovakia; SWE, Sweden; SYR, Syria; TUR, Turkey; UKR, Ukraine; YUG, Yugoslavia, ZAF, South Africa. ‘Latitude and longitude’ are given in decimal degrees. Accessions supplied without detailed information on sampling location are assigned latitude and longitude representing the country of origin (National Geospatial Intelligence Agency ‘Country Coordinates’: http://earth-info.nga.mil/gns/html/gis_countryfiles.htm) and the coordinates for these accessions are italicised. ‘Source’ shows the germplasm collection supplying the accession using the following abbreviations: CRA, Centre Researches de Agricole de Gembloux, Gembloux, Belgium; INRA, l’Institut National de la Recherche Agronomique, Paris, France; IPK, Institut für Pflanzengenetik und Kulturpflanzenforschung, Gatersleben, Germany; INIA, Instituto Nacional de Investigación y Tecnología Agraria y Alimentaria, Madrid, Spain; JIC, Institute of Plant Science Research Collection of Wheat and Related Species, John Innes Centre, Norwich, UK; NGB, Nordic Gene Bank, Alnarp, Sweden; NSGC, National Small Grains Collection, Idaho, USA; RAC, Station de Recherche Agroscope Changins, Nyon, Switzerland; RCAT, Research Centre for Agrobotany, Tápiószele, Hungary; SCRI, J. Russell, Scottish Crops Research Institute, Dundee, UK; VAV, N.I. Vavilov Institute of Plant Industry, 42–44, B. Morskaya Street, 190000, St. Petersburg, Russia.

Table S1. Barley accessions used in this study

| Source Number | Collection Site | Country | Latitude | Longitude | Source |
| --- | --- | --- | --- | --- | --- |
| HOR 378 | Russe | BGR | 43.86 | 25.97 | IPK |
| HOR 396 | Burgas | BGR | 42.50 | 27.47 | IPK |
| HOR 676 | Kreta / Lukia | GRC | 34.98 | 25.02 | IPK |
| HOR 683 | Peloponnes / Prassia | GRC | 39.19 | 21.49 | IPK |
| HOR 684 | Peloponnes / Lirkia | GRC | 37.70 | 22.55 | IPK |
| HOR 686 | Peloponnes / Aj. Giorgitika near Tripolis | GRC | 37.51 | 22.38 | IPK |
| HOR 691 | Peloponnes / Pyrgos | GRC | 37.68 | 21.45 | IPK |
| HOR 697 | Peloponnes / Panidsa | GRC | 36.75 | 22.45 | IPK |
| HOR 699 | Peloponnes / Mavrowunion | GRC | 36.73 | 22.57 | IPK |
| HOR 700 | Peloponnes / Polowitsa | GRC | 36.93 | 22.47 | IPK |
| HOR 703 | Peloponnes / Selina | GRC | 39.19 | 21.11 | IPK |
| HOR 707 | Peloponnes / Stephanias | GRC | 39.26 | 21.48 | IPK |
| HOR 713 | Peloponnes / Afisu near Sparta | GRC | 38.03 | 23.17 | IPK |
| HOR 722 | Peloponnes / Musaki near Sparta | GRC | 38.03 | 23.17 | IPK |
| HOR 726 | Peloponnes / Longa | GRC | 36.87 | 21.90 | IPK |
| HOR 738 | N Greece / Kavaje | GRC | 41.05 | 24.58 | IPK |
| HOR 742 | N Greece / Kepesowon | GRC | 39.89 | 20.78 | IPK |
| HOR 743 | N Greece / Tsepelowon | GRC | 39.91 | 20.82 | IPK |
| HOR 760 | N Greece / Neokhori | GRC | 39.07 | 21.02 | IPK |
| HOR 764 | N Greece / Asfaka | GRC | 39.78 | 20.75 | IPK |
| HOR 768 | N Greece / Aspranyeli | GRC | 39.82 | 20.73 | IPK |
| HOR 850 | Kreta / Sitia | GRC | 35.20 | 26.10 | IPK |
| HOR 862 | Kreta / Topolia - Kaludiana, between | GRC | 35.43 | 23.68 | IPK |
| HOR 863 | Kreta / Lassithi plateau | GRC | 35.08 | 25.83 | IPK |
| HOR 865 | Kreta / Palaeochora, W | GRC | 35.23 | 23.68 | IPK |
| HOR 868 | Kreta / Lakki | GRC | 37.13 | 26.85 | IPK |
| HOR 873 | Burgas | BGR | 42.50 | 27.47 | IPK |
| HOR 936 | N Greece / Skamneli | GRC | 39.91 | 20.85 | IPK |
| HOR 966 | N Greece / Ptolomais, N | GRC | 40.51 | 21.68 | IPK |
| HOR 982 | N Greece / Ardea | GRC | 40.97 | 22.06 | IPK |
| HOR 986 | N Greece / Xinon Neron | GRC | 40.68 | 21.62 | IPK |
| HOR 987 | N Greece / Perdika, N | GRC | 39.37 | 20.30 | IPK |
| HOR 988 | N Greece / Ptolomais, N | GRC | 40.51 | 21.68 | IPK |
| HOR 990 | N Greece / Wathilakkos | GRC | 40.77 | 22.70 | IPK |
| HOR 991 | N Greece / Janina, Agr. Exp. Stn. | GRC | 39.67 | 20.85 | IPK |
| HOR 994 | Peloponnes / Karteri | GRC | 37.85 | 22.42 | IPK |
| HOR 995 | Peloponnes / Drisa | GRC | 40.83 | 24.20 | IPK |
| HOR 1007 | N Greece / Nea Anchialios | GRC | 39.28 | 22.82 | IPK |
| HOR 1015 | Peloponnes / Pikerni | GRC | 37.65 | 22.42 | IPK |
| HOR 1025 | Peloponnes / Tsakona | GRC | 37.27 | 23.10 | IPK |
| HOR 1145 | Peloponnes / Dimitsana | GRC | 37.59 | 22.04 | IPK |
| HOR 1150 | Peloponnes / Lesi | GRC | 37.53 | 23.28 | IPK |
| HOR 1168 | Peloponnes / Karteri | GRC | 37.85 | 22.42 | IPK |
| HOR 1233 | N Greece / Ardea | GRC | 40.97 | 22.06 | IPK |
| HOR 1256 | Kreta / Erimopolis | GRC | 35.27 | 26.28 | IPK |
| HOR 1276 | N Greece / Kukuli | GRC | 40.88 | 22.94 | IPK |
| HOR 1283 | N Greece / Platikambos | GRC | 39.62 | 22.53 | IPK |
| HOR 1285 | Radomir | ALB | 41.82 | 20.48 | IPK |
| HOR 1379 | Kreta / Elos | GRC | 35.37 | 23.63 | IPK |
| HOR 1386 | N Greece / Skamneli | GRC | 39.91 | 20.85 | IPK |
| HOR 1652 | Peloponnes / Analipsis | GRC | 37.80 | 21.43 | IPK |
| HOR 1659 | Peloponnes / Karteri | GRC | 37.85 | 22.42 | IPK |
| HOR 1668 | Cerasi, Provinz Reggio Calabria, Westseite des Aspromonte, Süditalien | ITA | 38.17 | 15.75 | IPK |
| HOR 1669 | Domanico, südwestlich von Cosenza, Provinz Cosenza, Süditalien | ITA | 39.22 | 16.20 | IPK |
| HOR 1670 | Consorzio Agrario Cosenza, Süditalien | ITA | 39.30 | 16.25 | IPK |
| HOR 1770 | Kreta / Palaeochora | GRC | 35.23 | 23.68 | IPK |
| HOR 1773 | Kreta, Wutas | GRC | 35.30 | 23.65 | IPK |
| HOR 1943 | Peloponnes / Bissia | GRC | 38.02 | 22.98 | IPK |
| HOR 2000 | Palinuro (Marina), Provinz Salerno, Süditalien | ITA | 40.03 | 15.28 | IPK |
| HOR 2002 | Gioiosa Superiore Ionica, Provinz Reggio Calabria, Süditalien | ITA | 38.33 | 16.30 | IPK |
| HOR 2003 | Ardore Marina, Provinz Reggio Calabria, Süditalien | ITA | 38.17 | 16.20 | IPK |
| HOR 2004 | Gallodoro, Provinz Messina, Süditalien | ITA | 37.90 | 15.30 | IPK |
| HOR 2006 | bei Crotone, Provinz Catanzaro, Süditalien | ITA | 39.08 | 17.13 | IPK |
| HOR 2007 | unterhalb von Caccuri am Neto, Provinz Catanzaro, Süditalien | ITA | 39.23 | 16.78 | IPK |
| HOR 2008 | Sila, San Giovanni in Fiore, Provinz Cosenza, Süditalien | ITA | 39.25 | 16.70 | IPK |
| HV0036 | Laax | CHE | 46.80 | 9.25 | RAC |
| HV0037 | Laax | CHE | 46.80 | 9.25 | RAC |
| HV0039 | Laax | CHE | 46.80 | 9.25 | RAC |
| HV0040 | Laax | CHE | 46.80 | 9.25 | RAC |
| HV0041 | Fellers | CHE | 46.80 | 9.22 | RAC |
| HV0042 | Fellers | CHE | 46.80 | 9.22 | RAC |
| HV0043 | Fellers | CHE | 46.80 | 9.22 | RAC |
| HV0063 | Vrin | CHE | 46.65 | 9.10 | RAC |
| HV0065 | Vrin | CHE | 46.65 | 9.10 | RAC |
| HV0066 | Vrin | CHE | 46.65 | 9.10 | RAC |
| HV0067 | Vrin | CHE | 46.65 | 9.10 | RAC |
| HV0068 | Vrin | CHE | 46.65 | 9.10 | RAC |
| HV0079 | Fellers | CHE | 46.80 | 9.22 | RAC |
| HV0080 | Fellers | CHE | 46.80 | 9.22 | RAC |
| HV0082 | Ladir | CHE | 46.80 | 9.20 | RAC |
| HV0083 | Ladir | CHE | 46.80 | 9.20 | RAC |
| HV0084 | Ladir | CHE | 46.80 | 9.20 | RAC |
| HV0108 | Lussai | CHE | 46.61 | 10.38 | RAC |
| HV0109 | Lussai | CHE | 46.61 | 10.38 | RAC |
| HV0110 | Lussai | CHE | 46.61 | 10.38 | RAC |
| HV0111 | Lussai | CHE | 46.61 | 10.38 | RAC |
| HV0112 | St Maria | CHE | 46.60 | 10.43 | RAC |
| HV0113 | St Maria | CHE | 46.60 | 10.43 | RAC |
| HV0114 | St Maria | CHE | 46.60 | 10.43 | RAC |
| HV0151 | Vex | CHE | 46.20 | 7.40 | RAC |
| HV0152 | Vex | CHE | 46.20 | 7.40 | RAC |
| HV0153 | Vex | CHE | 46.20 | 7.40 | RAC |
| HV0154 | Vex | CHE | 46.20 | 7.40 | RAC |
| HV0242 | Biel | CHE | 47.17 | 7.25 | RAC |
| HV0243 | Biel | CHE | 47.17 | 7.25 | RAC |
| HV0244 | Biel | CHE | 47.17 | 7.25 | RAC |
| HV0245 | Biel | CHE | 47.17 | 7.25 | RAC |
| HV0262 | Geschinen | CHE | 46.48 | 8.28 | RAC |
| HV0263 | Geschinen | CHE | 46.48 | 8.28 | RAC |
| HV0264 | Geschinen | CHE | 46.48 | 8.28 | RAC |
| HV0265 | Geschinen | CHE | 46.48 | 8.28 | RAC |
| HV0266 | Geschinen | CHE | 46.48 | 8.28 | RAC |
| HV0267 | Geschinen | CHE | 46.48 | 8.28 | RAC |
| HV0297 | Sariese | CHE | 46.25 | 7.35 | RAC |
| HV0298 | Sariese | CHE | 46.25 | 7.35 | RAC |
| HV0503 | Feldis | CHE | 46.78 | 9.43 | RAC |
| HV0508 | Siat | CHE | 46.79 | 9.16 | RAC |
| HV0521 | Brigels | CHE | 46.77 | 9.07 | RAC |
| HV0522 | Brigels | CHE | 46.77 | 9.07 | RAC |
| HV0524 | Brigels | CHE | 46.77 | 9.07 | RAC |
| HV0553 | Rueras | CHE | 46.75 | 8.68 | RAC |
| HV0555 | Rueras | CHE | 46.75 | 8.68 | RAC |
| HV0556 | Rueras | CHE | 46.75 | 8.68 | RAC |
| HV0573 | Platta | CHE | 46.65 | 8.85 | RAC |
| HV0574 | Platta | CHE | 46.65 | 8.85 | RAC |
| HV0575 | Meierhof | CHE | 46.75 | 9.10 | RAC |
| HV0576 | Meierhof | CHE | 46.75 | 9.10 | RAC |
| HV0577 | Meierhof | CHE | 46.75 | 9.10 | RAC |
| HV0579 | Meierhof | CHE | 46.75 | 9.10 | RAC |
| HV0581 | Misanenga | CHE | 46.75 | 9.12 | RAC |
| HV0582 | Misanenga | CHE | 46.75 | 9.12 | RAC |
| HV0583 | Misanenga | CHE | 46.75 | 9.12 | RAC |
| HV0585 | Fuormis | CHE | 46.64 | 8.85 | RAC |
| HV0587 | Platta | CHE | 46.65 | 8.85 | RAC |
| HV0588 | Platta | CHE | 46.65 | 8.85 | RAC |
| HV0591 | Susch | CHE | 46.75 | 10.08 | RAC |
| HV0592 | Susch | CHE | 46.75 | 10.08 | RAC |
| HV0595 | Lavin | CHE | 46.77 | 10.10 | RAC |
| HV0596 | Lavin | CHE | 46.77 | 10.10 | RAC |
| HV0597 | Lavin | CHE | 46.77 | 10.10 | RAC |
| HV0599 | Giarsun | CHE | 46.77 | 10.14 | RAC |
| HV0600 | Giarsun | CHE | 46.77 | 10.14 | RAC |
| HV0601 | Giarsun | CHE | 46.77 | 10.14 | RAC |
| HV0602 | Zernez | CHE | 46.70 | 10.10 | RAC |
| HV0603 | Zernez | CHE | 46.70 | 10.10 | RAC |
| HV0604 | Guarda | CHE | 46.78 | 10.15 | RAC |
| HV0605 | Guarda | CHE | 46.78 | 10.15 | RAC |
| HV0606 | Guarda | CHE | 46.78 | 10.15 | RAC |
| HV0611 | Plan | CHE | 46.88 | 7.08 | RAC |
| HV0645 | Schuls | CHE | 46.80 | 10.30 | RAC |
| HV0653 | Fetan | CHE | 46.80 | 10.23 | RAC |
| HV0655 | Fetan | CHE | 46.80 | 10.23 | RAC |
| HV0656 | Fetan | CHE | 46.80 | 10.23 | RAC |
| HV0665 | Tiefencastel | CHE | 46.65 | 9.58 | RAC |
| HV0666 | Tiefencastel | CHE | 46.65 | 9.58 | RAC |
| HV0667 | Tiefencastel | CHE | 46.65 | 9.58 | RAC |
| HV0668 | Tiefencastel | CHE | 46.65 | 9.58 | RAC |
| HV0693 | Patzen | CHE | 46.64 | 9.43 | RAC |
| HV0695 | Patzen | CHE | 46.64 | 9.43 | RAC |
| HV0696 | Patzen | CHE | 46.64 | 9.43 | RAC |
| HV0699 | Patzen | CHE | 46.64 | 9.43 | RAC |
| HV0700 | Patzen | CHE | 46.64 | 9.43 | RAC |
| HV0701 | Patzen | CHE | 46.64 | 9.43 | RAC |
| HV0704 | Patzen | CHE | 46.64 | 9.43 | RAC |
| HV0705 | Patzen | CHE | 46.64 | 9.43 | RAC |
| HV0709 | Affeier | CHE | 46.76 | 9.12 | RAC |
| HV0712 | Valata | CHE | 46.76 | 9.13 | RAC |
| HV0714 | Valata | CHE | 46.76 | 9.13 | RAC |
| HV0717 | Luven | CHE | 46.77 | 9.18 | RAC |
| HV0718 | Luven | CHE | 46.77 | 9.18 | RAC |
| HV0721 | Tersnaus | CHE | 46.69 | 9.18 | RAC |
| HV0722 | Tersnaus | CHE | 46.69 | 9.18 | RAC |
| 9327 | Professeur Damseaux Sélection dans une population de pays du Condroz (Belgique) | BEL | *50.33* | *5.00* | INRA |
| 9367 | Hatif De Grignon Sélection dans une population de pays de l'Ile de Ré | FRA | *46.20* | *-1.37* | INRA |
| 9434 | Kirgizskij 247 Selection Dans Une Population De Pays De Kirghizie | UKR | *47.65* | *34.80* | INRA |
| 9443 | Krasnyj Dar~2494 Selection Dans Une Population De Pays De Krasnodar,USR | RUS | *45.03* | *38.98* | INRA |
| 9480 | Mozdokski (13099) Sélection dans une population de pays d'Ossétie du Nord | RUS | *44.03* | *44.73* | INRA |
| 9775 | Derenburger Selection Dans Une Population De Pays De Almersfelder | DEU | *51.43* | *8.67* | INRA |
| 9784 | Urgel Variété de pays catalane | ESP | *41.42* | *0.60* | INRA |
| 9799 | Villardefrade De Campos Variété de pays | ESP | *41.72* | *-5.25* | INRA |
| 9938 | Escourgeon Extra Hatif Sélection dans une Variété de pays de Vendée | FRA | *46.67* | *-1.33* | INRA |
| 9939 | Hatif Du Douaisis Sélection dans une population du Douaisis | FRA | *50.37* | *3.08* | INRA |
| 9982 | Black Russian Variété de pays de Tiflis; gène Mla2 | GEO | *41.72* | *44.78* | INRA |
| 10078 | Barley-Spratt Sélection dans orge de pays | GBR | *53.00* | *-2.00* | INRA |
| 10085 | Italien. Nackte Variété d'orge italienne ancienne ( éthiopienne?) | ITA | *42.83* | *12.83* | INRA |
| 10682 | Bigo Selection Dans Une Population De Pays De Zelande,Nld | NLD | *51.42* | *3.75* | INRA |
| 10683 | Binder Selection Dans Hanna | DNK | *56.00* | *10.00* | INRA |
| 10696 | Brehat Sélection dans une population de l'île de Brehat | FRA | *48.85* | *-3.00* | INRA |
| 10706 | C79 Paumelle Sélection dans une Population de pays du Gard - Castelnaudary | FRA | *43.32* | *1.95* | INRA |
| 10707 | C80 Paumelle Population De Pays Du Var | FRA | *43.50* | *6.33* | INRA |
| 10816 | CI5638-7 Variété de pays d'Otrada Kubanska | RUS | *45.24* | *40.83* | INRA |
| 10876 | Decorticatum Collecte d'origine inconnue | NLD | *53.00* | *5.00* | INRA |
| 10888 | Dickson 628 Variété de pays de la région de Kharkhov | UKR | *50.00* | *36.25* | INRA |
| 10963 | FP2 Sélection dans une variété de pays du Finistère | FRA | *48.33* | *-4.00* | INRA |
| 11476 | Noire A Balles Panachees Orge exotique ancienne | FRA | *46.00* | *2.00* | INRA |
| 11488 | O144 Sélection dans une variété de pays de Mayenne | FRA | *48.08* | *-0.67* | INRA |
| 11526 | Pflugs Intensiv Sélection dans une orge de pays Sarroise | DEU | *49.40* | *6.96* | INRA |
| 11609 | Souche 142 De Colmar Sélection dans une population de pays de Statzheim | FRA | *48.38* | *7.48* | INRA |
| 11611 | Souche 191 Sélection dans une population de pays du Haut-Rhin | FRA | *48.00* | *7.33* | INRA |
| 11805 | WSN Weihenstephaner Schwarzer Nackte | DEU | *48.50* | *11.50* | INRA |
| 11914 | Cervecera De Burquete Variété de pays de Navarre | ESP | *43.00* | *-1.50* | INRA |
| 12112 | E71 Oisans Variété De Pays De L'oisans | FRA | *45.03* | *6.03* | INRA |
| 12248 | Choche | NLD | *53.10* | *5.10* | INRA |
| 12346 | Old Cornish Variété de Pays de Cornouailles | GBR | *50.42* | *-4.75* | INRA |
| 12347 | Hen Gymro Variété de pays du Pays de Galles | GBR | *52.50* | *-3.50* | INRA |
| 12348 | Archer selection dans des orges de l'est et du sud de L'angleterre | GBR | *51.25* | *-1.92* | INRA |
| 12349 | Chevalier selection dans une population de pays de Debenham | GBR | *52.23* | *1.18* | INRA |
| 12453 | Suede 1 Sélection dans une orge suédoise | SWE | *62.00* | *15.00* | INRA |
| 12491 | Epeautree Sélectiuon dans une variété population de pays | FRA | *46.00* | *2.00* | INRA |
| 12493 | D'assas Sélection dans une population de pays du Gâtinais | FRA | *48.00* | *2.33* | INRA |
| 12494 | Alsace 121 Sélection dans une variété population de pays d'Alsace | FRA | *48.50* | *7.50* | INRA |
| 12495 | Alsace 104 Sélection dans une variété population de pays d'Alsace | FRA | *48.50* | *7.50* | INRA |
| 12496 | Alsace 75 Sélection dans une variété population de pays d'Alsace | FRA | *48.50* | *7.50* | INRA |
| 12504 | Premiere A Barbes Lisses Variété exotique ancienne d'origine indéterminée | FRA | *46.00* | *2.00* | INRA |
| 12509 | Souche 106 De Colmar Sélection dans une variété de pays de Gertwiller | FRA | *48.40* | *7.47* | INRA |
| 12511 | Goldthorpe trouve en Angleterre dans un champ d'orge Chevallier | GBR | *53.53* | *-1.30* | INRA |
| 12512 | Le Puy N11 Sélection dans une variété de pays d'Auvergne (Le Puy - Haute-Loire) | FRA | *45.08* | *3.83* | INRA |
| 12513 | Le Puy N12 Sélection dans une variété de pays d'Auvergne (Le Puy - Haute-Loire) | FRA | 45.08 | 3.83 | INRA |
| 12583 | It Nackte Italian Nackte | ITA | *42.83* | *12.83* | INRA |
| 12587 | Orge Du Ronzet Sélection dans une population de pays de Haute-Loire | FRA | *45.73* | *2.62* | INRA |
| 12607 | Bavaria Sélection dans une variété de pays de Basse-Bavière | DEU | *48.50* | *11.50* | INRA |
| 12609 | Nue Noire Du Prophete Orge exotique ancienne | FRA | *46.00* | *2.00* | INRA |
| 12698 | Orge Eventail Orge exotique ancienne | FRA | *46.00* | *2.00* | INRA |
| 12970 | Chautignac Variété de pays de Chautignac près de Murols, Puy-de Dôme; | FRA | 45.58 | 2.95 | INRA |
| 13202 | Noire Deux Rangs Montpellier Variété de pays ancienne | FRA | 43.60 | 3.88 | INRA |
| 13855 | Nue De Coire Variété de pays ancienne des Grisons | CHE | *46.85* | *9.50* | INRA |
| 4443 | Ambeluzos, Kreta | GRC | 35.07 | 24.92 | JIC |
| 7371 | Pallidum 45 Knezha Sartov | BGR | 43.50 | 24.08 | JIC |
| 20561 | Bere: Burland, Tronda | GBR | *60.42* | *-1.32* | JIC |
| PI 22493 | Libochovice, North Bohemia | CZE | *50.40* | *14.03* | NSGC |
| PI 5590 | Kitzingen, Bavaria | DEU | *49.73* | *10.17* | NSGC |
| PI 5846 | Binsbach, near Gonheim, Bavaria | DEU | *49.95* | *10.00* | NSGC |
| PI 5851 | Klausberg, Bavaria | DEU | *49.65* | *11.43* | NSGC |
| PI 5853 | Oberroning, | GBR | 48.73 | 12.08 | NSGC |
| PI 5873 | Essleben, Bavaria | DEU | 49.95 | 10.08 | NSGC |
| PI 40647 | Alanas parsonage, Jamtland | SWE | 64.17 | 15.70 | NSGC |
| PI 54912 | Opdal, central high-mountain region, Sor-Trondelag | NOR | 62.50 | 9.67 | NSGC |
| PI 54915 | Donnes, Nordland, Nordland | NOR | 66.20 | 12.58 | NSGC |
| CIho 5003 | County Donegal, Donegal | IRL | 54.92 | -8.00 | NSGC |
| PI 94841 | Russia | RUS | *42.00* | *45.00* | NSGC |
| PI 94843 | Russia | RUS | *42.00* | *45.00* | NSGC |
| PI 94845 | Russia | RUS | *42.00* | *45.00* | NSGC |
| PI 94846 | Russia | RUS | *45.00* | *35.00* | NSGC |
| PI 94847 | near Bristol, England From: Former Soviet Union | RUS | *45.20* | *35.20* | NSGC |
| PI 130740 | Hoy, Orkney Islands, Orkney Islands | GBR | 58.85 | -3.30 | NSGC |
| PI 183631 | Aspang, Lower Austria | AUT | 47.57 | 16.10 | NSGC |
| PI 183632 | Horn, Lower Austria | AUT | 48.67 | 15.67 | NSGC |
| PI 183635 | Ramsau, Lower Austria | AUT | 48.00 | 15.80 | NSGC |
| PI 183636 | Stockerau, Lower Austria | AUT | 48.40 | 16.22 | NSGC |
| PI 184050 | Novo Kikinda, Serbia | YUG | 45.83 | 20.47 | NSGC |
| PI 184051 | Zrenjanin, Serbia | YUG | 45.33 | 20.33 | NSGC |
| PI 184052 | Velika Kikinda, Serbia | YUG | 45.82 | 20.45 | NSGC |
| PI 184053 | Velika Kikinda, Serbia | YUG | 45.82 | 20.45 | NSGC |
| PI 184054 | Velika Kikinda, Serbia | YUG | 45.82 | 20.45 | NSGC |
| PI 184055 | Novo Becej, Serbia | YUG | 45.60 | 20.13 | NSGC |
| PI 184056 | Backa Palanka, Serbia | YUG | 45.25 | 19.38 | NSGC |
| PI 184057 | Backa Palanka, Serbia | YUG | 45.25 | 19.38 | NSGC |
| PI 184058 | Stari Sivac, Serbia | YUG | 45.70 | 19.37 | NSGC |
| PI 184059 | Srbobran, Serbia | YUG | 45.53 | 19.83 | NSGC |
| PI 184061 | Irig, Serbia | YUG | 45.10 | 19.87 | NSGC |
| PI 184062 | Ilok, Vukovarsko-srijemska | HRV | 45.22 | 19.37 | NSGC |
| PI 184071 | Osijek, Osjecko-baranjska zu | HRV | 45.55 | 18.68 | NSGC |
| PI 184072 | Kaptol, Pozesko-slavonska zu | HRV | 45.43 | 17.72 | NSGC |
| PI 184082 | Donji Lapac, Serbia | YUG | 44.88 | 19.10 | NSGC |
| PI 184083 | Travnik, Serbia | YUG | 44.78 | 19.65 | NSGC |
| PI 184084 | Livno, | BOS | 43.82 | 17.00 | NSGC |
| PI 636056 | Bodrogolaszi, Borsod-Abauj-Zemplen | MAG | 48.30 | 21.53 | NSGC |
| PI 636058 | Komadi, Hajdu-Bihar | MAG | 47.02 | 21.53 | NSGC |
| PI 636063 | Marcalto, Veszprem | MAG | 47.43 | 17.35 | NSGC |
| PI 636066 | Oroshaza, Bekes | MAG | 46.55 | 20.67 | NSGC |
| HOR 2236 | Sanvobor | ROM | 46.00 | 25.00 | IPK |
| HOR 2278 | N Greece / Buchunista - Megalochol | GRC | 39.56 | 21.84 | IPK |
| HOR 2281 | Peloponnes / Bissia | GRC | 38.02 | 22.98 | IPK |
| HOR 2285 | Mongiuffi, Provinz Messina, Süditalien | ITA | 37.92 | 15.27 | IPK |
| HOR 2346 | Tirol / Gries im Sulztal | AUT | 47.07 | 11.02 | IPK |
| HOR 2348 | Tirol / Sulztal | AUT | 47.07 | 11.02 | IPK |
| HOR 2392 | Osten der Sila, Longobucco, Provinz Cosenza, Süditalien | ITA | 39.25 | 16.50 | IPK |
| HOR 2404 | Griesi i. Sellrain | AUT | 47.20 | 11.15 | IPK |
| HOR 2414 | Calanna, Provinz Reggio Calabria, Westseite des Aspromonte, Süditalien | ITA | 38.18 | 15.72 | IPK |
| HOR 2486 | Osten der Sila, Longobucco, Provinz Cosenza, Süditalien | ITA | 39.25 | 16.50 | IPK |
| HOR 2510 | N Greece / Kepesowon | GRC | 39.89 | 20.78 | IPK |
| HOR 2674 | Kreta / Wukolis and Anoskoli, between | GRC | 35.47 | 23.80 | IPK |
| HOR 2719 | Sonsonate | SLV | 46.25 | 15.17 | IPK |
| HOR 2901 | Sonsonate | SLV | 46.25 | 15.17 | IPK |
| HOR 3073 | Stavropol' | RUS | 45.04 | 41.97 | IPK |
| HOR 3098 | Kabardino-Balkarskaja ASSR | RUS | 43.50 | 43.50 | IPK |
| HOR 3259 | Krasnodar | RUS | 45.03 | 38.98 | IPK |
| HOR 3260 | Krasnodar | RUS | 45.03 | 38.98 | IPK |
| HOR 3261 | Stavropol' | RUS | 45.04 | 41.97 | IPK |
| HOR 3262 | Zakarpatskaja Oblast | UKR | 48.33 | 23.00 | IPK |
| HOR 3263 | Stanislavskaja Oblast | SUN | 55.29 | 161.25 | IPK |
| HOR 7381 | Hrcava, östl. des Jablunka Passes, Mährisch-Schlesische Beskiden | CZE | 49.52 | 18.75 | IPK |
| HOR 7385 | Hutisko Solanec, Vsetínské Vrchy NW Vel. Karlovice | CZE | 49.43 | 18.22 | IPK |
| HOR 7387 | Hutisko Solanec, Vsetínské Vrchy NW Vel. Karlovice | CZE | 49.43 | 18.22 | IPK |
| HOR 7390 | Muránska Zdychava nördlich Revúca, Slowakisches Erzgebirge | SVK | 48.73 | 20.15 | IPK |
| HOR 7391 | Matiaska, ONO von Presov | SVK | 49.07 | 21.58 | IPK |
| HOR 7392 | Sobotiste, Weiße Karpaten | SVK | 48.72 | 17.40 | IPK |
| HOR 7393 | Tisovské Lazy | SVK | 48.68 | 19.95 | IPK |
| HOR 7418 | Beskid Sadecki / Sucha Dolina | POL | 52.00 | 19.10 | IPK |
| HOR 7428 | Leningrad | RUS | 59.89 | 30.26 | IPK |
| HOR 7436 | Klubina, Rand der Slowakischen Beskiden | SVK | 49.35 | 18.90 | IPK |
| HOR 7437 | Klubina, Rand der Slowakischen Beskiden | SVK | 49.35 | 18.90 | IPK |
| HOR 7438 | Hutisko Solanec, Vsetínské Vrchy NW Vel. Karlovice | CZE | 49.43 | 18.22 | IPK |
| HOR 7519 | Beskid Sadecki / Sucha Dolina | POL | 52.00 | 19.10 | IPK |
| RCAT008634 | Balatonfôkajár | MAG | 47.02 | 18.22 | RCAT |
| RCAT007250 | Békéscsaba | MAG | 46.68 | 21.10 | RCAT |
| RCAT008648 | Emôd | MAG | 47.93 | 20.82 | RCAT |
| RCAT008640 | Geszt | MAG | 46.88 | 21.58 | RCAT |
| RCAT008653 | Kerta | MAG | 47.17 | 17.28 | RCAT |
| RCAT008637 | Kisberény | MAG | 46.63 | 17.67 | RCAT |
| RCAT008642 | Komádi | MAG | 47.00 | 21.50 | RCAT |
| RCAT008651 | Marcaltô | MAG | 47.43 | 17.37 | RCAT |
| RCAT008659 | Orosháza | MAG | 46.57 | 20.67 | RCAT |
| RCAT008646 | Ravazd | MAG | 47.52 | 17.75 | RCAT |
| RCAT009558 | Tápiósüly | MAG | 47.45 | 19.55 | RCAT |
| RCAT008649 | Écs | MAG | 47.55 | 17.72 | RCAT |
| RCAT009815 | ujszász | MAG | 47.30 | 20.08 | RCAT |
| 27 | Sarkalahti ME0103 Sarkalahti, Luum | FIN | 61.03 | 27.33 | NGB |
| 792 | Luusua EH0401 Luusua, Kemijärv | FIN | 66.48 | 27.35 | NGB |
| 1158 | Leskelä ME0102 Leskelä, Piippol | FIN | 64.18 | 25.82 | NGB |
| 1159 | Kilpau ME0201; 4RW D Kilpau, Oulainen | FIN | 64.33 | 25.20 | NGB |
| 2784 | Långstrand 0102; Paavo Mix Långstrand, Kors | FIN | 63.03 | 21.90 | NGB |
| 2812 | Valtaila 0301; Mari Mix Valtaila, Isokyr | FIN | 62.97 | 22.43 | NGB |
| 775 | Allsån ME0401 Överkalix, Allså | SWE | 66.40 | 22.93 | NGB |
| 776 | Överkalix PH0301 Överkalix | SWE | 66.40 | 22.77 | NGB |
| 259 | Järvenkylä ME0302 SEP B cereal mix Järvenkylä, Mieh | FIN | 60.72 | 27.48 | NGB |
| 785 | Sattanen EH0103 Sattanen, Sodank | FIN | 67.58 | 26.62 | NGB |
| 773 | Erkheikki PH0101 Erkheikki | SWE | 67.23 | 23.22 | NGB |
| HOR 10221 | Fardella, Provinz Potenza, Region Basilicata | ITA | 40.12 | 16.17 | IPK |
| HOR 10223 | Castelluccio Superiore, Provinz Potenza, Region Basilicata, 8 km N des Ortes | ITA | 40.02 | 15.98 | IPK |
| HOR 10228 | 5 km no San Pietro in Guarano, Castanea-Waldgürtel, Provinz Cosenza, Region Calabria | ITA | 39.33 | 16.32 | IPK |
| HOR 10231 | Cuoggio del Cuoco bei San Pietro in Guarano, am Abkürzungsweg zur Hauptstraße nach Camigliatello, Provinz Cosenza, Region Calabria | ITA | 39.33 | 16.32 | IPK |
| HOR 10233 | Germano, 10 km nw San Giovanni in Fiore, Plateau im s des Silagrande-Gebirges, Provinz Cosenza, Region Calabria | ITA | 39.32 | 16.65 | IPK |
| HOR 10234 | Salinella, 4 km w Roccabernarda, Provinz Catanzaro, Region Calabria | ITA | 39.07 | 16.80 | IPK |
| HOR 10364 | Soveria Mannelli, Consorzio Agrario, Provinz Cosenza, Region Calabria | ITA | 39.08 | 16.37 | IPK |
| HOR 10366 | Chiaravalle Centrale, Provinz Catanzaro, Region Calabria | ITA | 38.68 | 16.42 | IPK |
| HOR 10367 | Rometta, Provinz Messina, Sizilien | ITA | 38.05 | 14.87 | IPK |
| HOR 10409 | Klembow | POL | 52.42 | 21.33 | IPK |
| HOR 10410 | Klembow | POL | 52.42 | 21.33 | IPK |
| HOR 10551 | Contrada Beata, zwischen S. Cataldo und Mussomeli, Sicilia | ITA | 37.58 | 13.75 | IPK |
| HOR 10553 | 5 km hinter Palazzo Adriano an der Straße nach Bisaquino, Sicilia | ITA | 37.70 | 13.25 | IPK |
| HOR 10554 | Fulgatore bei Trapani, Sig. Lemenza, lokaler Saatgutexperte, Sicilia | ITA | 38.02 | 12.48 | IPK |
| HOR 10555 | n von Ummari, Sicilia | ITA | 37.93 | 12.73 | IPK |
| HOR 10559 | zwischen Racalmuto und Milena, Sicilia | ITA | 37.40 | 13.73 | IPK |
| HOR 10630 | Rainbach (Mühlviertel) | AUT | 48.55 | 14.47 | IPK |
| HOR 10631 | Rosenhof (Mühlviertel) | AUT | 48.57 | 14.68 | IPK |
| HOR 10632 | Geierschlag | AUT | 48.50 | 14.85 | IPK |
| HOR 10635 | Klembow | POL | 52.42 | 21.33 | IPK |
| HOR 10638 | Contrada Soria, 4 km n von Mussomeli, Sicilia | ITA | 37.58 | 13.75 | IPK |
| HOR 10681 | SO-Kalabrien, S. Pantaleone | ITA | 37.98 | 15.87 | IPK |
| HOR 10682 | Ägadische Inseln, Insel Favignana | ITA | 37.93 | 12.32 | IPK |
| HOR 10772 | n von Ummari, Sicilia | ITA | 37.93 | 12.73 | IPK |
| HOR 10778 | Furci | ITA | 42.00 | 14.60 | IPK |
| HOR 10779 | Castiglione Messer Marino | ITA | 41.87 | 14.45 | IPK |
| HOR 10780 | zwischen Castiglione und Capracotta (8 km vor Capracotta) | ITA | 41.83 | 14.27 | IPK |
| HOR 10784 | Roccapia | ITA | 41.93 | 13.98 | IPK |
| HOR 10785 | Bisegna | ITA | 41.92 | 13.75 | IPK |
| HOR 10787 | Colle S. Martino | ITA | 45.90 | 12.08 | IPK |
| HOR 10788 | Contrada Turchi (Pizzoferrato) | ITA | 41.92 | 14.23 | IPK |
| HOR 10792 | 5 km vor Scanno | ITA | 41.90 | 13.88 | IPK |
| HOR 10936 | Capitignano, alte Mühle, Mittelitalien | ITA | 42.52 | 13.30 | IPK |
| HOR 10937 | 5 km nach Leonessa in Richtung Cascia, Mittelitalien | ITA | 42.57 | 12.97 | IPK |
| HOR 10941 | Camerino, Kapuzinerkloster, Mittelitalien | ITA | 43.13 | 13.07 | IPK |
| HOR 10942 | Camerino, Kapuzinerkloster, Mittelitalien | ITA | 43.13 | 13.07 | IPK |
| HOR 10943 | San Marino di Urbino, Mittelitalien | ITA | 44.62 | 11.42 | IPK |
| HOR 10944 | San Marino di Urbino, Mittelitalien | ITA | 44.62 | 11.42 | IPK |
| HOR 10945 | Scapezzano di Senigallia, Biodynamische Genossenschaft "La terra e il cielo", Mittelitalien | ITA | 43.72 | 13.15 | IPK |
| HOR 10946 | Secchiano, Mittelitalien | ITA | 43.93 | 12.32 | IPK |
| HOR 10947 | Podere Turricella bei Vessa (S. Pietro in Bagno), Mittelitalien | ITA | 43.90 | 12.02 | IPK |
| HOR 11003 | Region Emilia-Romagna, Porretta Terme (Bo.) | ITA | 44.15 | 10.98 | IPK |
| HOR 11004 | Region Emilia-Romagna, Casanova Pieve (Castel di Casio) (Bo.) | ITA | 44.17 | 11.03 | IPK |
| HOR 11005 | Region Trentino Alto Adige, San Valentino alla Muta (Bz.) | ITA | 46.77 | 10.53 | IPK |
| HOR 11006 | Region Trentino Alto Adige, Tubre (Bz.) | ITA | 46.65 | 10.45 | IPK |
| HOR 11008 | Region Trentino Alto Adige, Calice bei Vipiteno (Bz.) | ITA | 46.90 | 11.43 | IPK |
| HOR 11009 | Region Trentino Alto Adige, Dobbiaco (Bz.) | ITA | 46.74 | 12.23 | IPK |
| HOR 11012 | Capitignano, alte Mühle, Mittelitalien | ITA | 42.52 | 13.30 | IPK |
| HOR 11113 | Lugnano in Teverina (TR) | ITA | 42.57 | 12.33 | IPK |
| HOR 11114 | Bagnoregio (VT) - Poggiotono | ITA | 42.62 | 12.08 | IPK |
| HOR 11116 | Bruzolo (CN) | ITA | 44.38 | 7.53 | IPK |
| HOR 11117 | Bruzolo (CN) | ITA | 44.38 | 7.53 | IPK |
| HOR 11118 | Bruzolo (CN) | ITA | 44.38 | 7.53 | IPK |
| HOR 11119 | Roncaglia (CN) | ITA | 44.78 | 7.55 | IPK |
| HOR 11122 | Lugnano in Teverina (TR) | ITA | 42.57 | 12.33 | IPK |
| HOR 11123 | Astrio (Breno) BS | ITA | 45.95 | 10.30 | IPK |
| HOR 11124 | Astrio (Breno) BS | ITA | 45.95 | 10.30 | IPK |
| HOR 11125 | Roncaglia (CN) | ITA | 44.78 | 7.55 | IPK |
| HOR 12404 | Bogdan Voda, Izatal SW von Viseu de Sus, Kreis Maramures | ROM | 47.70 | 24.27 | IPK |
| HOR 12405 | Surduc, ca. 5 km NW von Buru, ca. 18 km SW von Turda, Muntii Apuseni | ROM | 46.53 | 23.55 | IPK |
| HOR 12406 | Agris, ca. 3,5 km N von Iara, ca. 20 km S von Cluj-Napoca, Muntii Apuseni | ROM | 46.58 | 23.52 | IPK |
| HOR 12407 | Baisoara, ca. 25 km SW von Cluj-Napoca, Muntii Apuseni | ROM | 46.58 | 23.47 | IPK |
| HOR 12408 | Muntele Baisorii, ca. 10 km W von Baisoara, ca. 30 km SW von Cluj-Napoca, Muntii Apuseni | ROM | 46.57 | 23.35 | IPK |
| HOR 12409 | Muntele Baisorii, ca. 10 km W von Baisoara, ca. 30 km SW von Cluj-Napoca, Muntii Apuseni | ROM | 46.57 | 23.35 | IPK |
| HOR 12410 | Matisesti, ca. 3 km O von Horea, Muntii Apuseni | ROM | 46.50 | 22.95 | IPK |
| HOR 12411 | Astrio (Breno) BS | ITA | 45.95 | 10.30 | IPK |
| HOR 12412 | Roncaglia (CN) | ITA | 44.78 | 7.55 | IPK |
| HOR 12721 | Bagnoregio (VT) - Poggiotono | ITA | 42.62 | 12.08 | IPK |
| HOR 12787 | Astrio (Breno) BS | ITA | 45.95 | 10.30 | IPK |
| HOR 7528 | Kotlina Sadecka / Olszanka W Stary Sacz | POL | 49.55 | 20.53 | IPK |
| HOR 7531 | Beskid Nizki - Bieszczady / Bukowsko | POL | 49.48 | 22.07 | IPK |
| HOR 7532 | Beskid Nizki - Bieszczady / Bukowsko | POL | 49.48 | 22.07 | IPK |
| HOR 7533 | Beskid Nizki - Bieszczady / Bukowsko | POL | 49.48 | 22.07 | IPK |
| HOR 7535 | Bieszczady, NW / Niebieszczany | POL | 49.52 | 22.17 | IPK |
| HOR 7537 | Bieszczady / Wolkowyja | POL | 49.33 | 22.42 | IPK |
| HOR 8675 | Inovec, südliches Vihorlat-Gebirge | SVK | 48.78 | 18.05 | IPK |
| HOR 8677 | Starina, Niedere Beskiden | SVK | 49.07 | 22.27 | IPK |
| HOR 8679 | Snina | SVK | 48.98 | 22.15 | IPK |
| HOR 8681 | Ruské, Niedere Beskiden | SVK | 49.12 | 22.35 | IPK |
| HOR 8685 | Beskid Wyspowy / Krasne Potockie, between Limanowa and Novi Sacz | POL | 49.67 | 20.58 | IPK |
| HOR 8702 | Palermo / Scanzano-Ficuzza | ITA | 37.88 | 13.37 | IPK |
| HOR 8808 | Kotlina Sandomierska / Cmolas, NNW Kolbuszowa | POL | 50.30 | 21.75 | IPK |
| HOR 8809 | Beskid Niski / Pobiedno, NE Bukowsko | POL | 49.53 | 22.12 | IPK |
| HOR 8810 | Beskid Niski / Pobiedno, NE Bukowsko | POL | 49.53 | 22.12 | IPK |
| HOR 8811 | Beskid Niski / Pobiedno, NE Bukowsko | POL | 49.53 | 22.12 | IPK |
| HOR 8813 | Beskid Niski / Pastwiska, SE Odrzechowa | POL | 49.53 | 21.93 | IPK |
| HOR 8814 | Beskid Niski / Glebokie, SE von Rymanow | POL | 49.55 | 21.92 | IPK |
| HOR 8815 | Beskid Niski / Rowne, NNE Dukla | POL | 49.58 | 21.72 | IPK |
| HOR 8819 | Beskid Niski/Sadecki / Florynka SSE Grybow | POL | 49.57 | 20.98 | IPK |
| HOR 8821 | Beskid Niski/Sadecki / Florynka E, SSE Grybow | POL | 49.57 | 20.98 | IPK |
| HOR 8823 | Beskid Niski/Sadecki / Florynka SSE Grybow | POL | 49.57 | 20.98 | IPK |
| HOR 8827 | Beskid Wyspowy / Krasne Potockie, Limanowa - Nowy Sacz | POL | 49.67 | 20.58 | IPK |
| HOR 8828 | Beskid Wyspowy / Krasne Potockie, Limanowa - Nowy Sacz | POL | 49.67 | 20.58 | IPK |
| HOR 8830 | Beskid Wyspowy / Slopnice W Limanowa | POL | 49.70 | 20.35 | IPK |
| HOR 8831 | Podhale Region / Lapszanka S Lapsze Wyzne | POL | 49.37 | 20.20 | IPK |
| HOR 8832 | Podhale Region / Lapszanka S Lapsze Wyzne | POL | 49.37 | 20.20 | IPK |
| HOR 8841 | Villanueve de los Castillejos | ESP | 37.50 | -7.27 | IPK |
| HOR 8842 | Valverde del Camino | ESP | 37.57 | -6.75 | IPK |
| HOR 8843 | Strasse Fregenal de la Sierra-Miguera la Real, 1 km von Higuera la Real, Badajoz | ESP | 38.13 | -6.68 | IPK |
| HOR 8844 | Fregenal de la Sierra | ESP | 38.17 | -6.65 | IPK |
| HOR 8845 | Strasse Fregenal de la Sierra-Miguera la Real, 1 km von Higuera la Real, Badajoz | ESP | 38.17 | -6.65 | IPK |
| HOR 8846 | Logrosan N | ESP | 39.33 | -5.48 | IPK |
| HOR 8849 | Montehermoso, Caceres | ESP | 40.08 | -6.35 | IPK |
| HOR 8850 | Aceituna | ESP | 40.15 | -6.33 | IPK |
| HOR 9247 | Pulinis, östlich Cisternino und NW von Carovigno (Puglia) | ITA | 40.73 | 17.42 | IPK |
| HOR 9252 | 4 km W Ostuni | ITA | 40.73 | 17.58 | IPK |
| HOR 9255 | 3 km südwestlich von Peschici | ITA | 41.95 | 16.02 | IPK |
| HOR 9256 | San Giorgio la Molara | ITA | 41.27 | 14.92 | IPK |
| HOR 9257 | Montefalcone | ITA | 43.73 | 10.73 | IPK |
| HOR 9258 | 2 km nördlich von Faeto, Azienda Marella Michele | ITA | 41.32 | 15.15 | IPK |
| HOR 9261 | 5 km südwestlich von San Marco in Lamis | ITA | 41.72 | 15.63 | IPK |
| HOR 9262 | 7 km von Vieste, an der Strasse nach Mattinata | ITA | 41.88 | 16.17 | IPK |
| HOR 9267 | Montefalcone | ITA | 43.73 | 10.73 | IPK |
| HOR 9272 | Ginestra (BN) | ITA | 40.93 | 15.73 | IPK |
| HOR 9274 | 1 km nördlich von Savignano Irpino | ITA | 41.23 | 15.18 | IPK |
| HOR 9275 | zwischen Monteleone di Puglia und Accadia | ITA | 41.17 | 15.25 | IPK |
| HOR 9277 | Beskid Nizki - Bieszczady / Bukowsko | POL | 49.48 | 22.07 | IPK |
| HOR 9278 | Beskid Nizki - Bieszczady / Bukowsko | POL | 49.48 | 22.07 | IPK |
| HOR 9450 | Cluj / Bedeciu | ROM | 46.80 | 23.13 | IPK |
| HOR 9451 | Cluj / Izvorul Crisului | ROM | 46.83 | 23.10 | IPK |
| HOR 9453 | Cluj / Capusul Mare | ROM | 46.78 | 23.30 | IPK |
| HOR 9674 | Kroviniaci, nördl. von Horná Súca, Biele Karpaty, Westhang | SVK | 48.97 | 17.98 | IPK |
| HOR 9702 | Fonte Greca | ITA | 41.45 | 14.18 | IPK |
| HOR 9704 | ö von Teora an der Strasse nach S. Andrea | ITA | 42.42 | 13.25 | IPK |
| HOR 9705 | ö von Teora an der Strasse nach S. Andrea | ITA | 42.42 | 13.25 | IPK |
| HOR 9708 | Castelgrande, innerhalb der Stadt | ITA | 40.78 | 15.43 | IPK |
| HOR 9712 | 2 km ö von Presenzano | ITA | 41.37 | 14.07 | IPK |
| HOR 9713 | SW von Nusco | ITA | 40.88 | 15.08 | IPK |
| HOR 9873 | 2 km w von S. Rufo in den Monti Alburni | ITA | 40.43 | 15.47 | IPK |
| HOR 9905 | District: Potenza, zwischen Casale und Ricigliano, 2,5 km vor Reach Ricigliano | ITA | 40.67 | 15.48 | IPK |
| HOR 9910 | District: Potenza, 4 km nö von Picerno an der Straße nach Li Foj bei einem öffentlichen Telefon | ITA | 40.63 | 15.63 | IPK |
| HOR 9911 | Contrada Pozzi, 5 km sö von Laurenzana auf einem großen Bauernhof | ITA | 40.47 | 15.97 | IPK |
|  | Ligne 129.9 | BEL | *50.83* | *4.00* | CRA |
|  | Ligne 14 | BEL | *50.83* | *4.00* | CRA |
|  | Ligne 185 | FRA | *46.67* | *-1.33* | CRA |
|  | Ligne 541 | BEL | *50.83* | *4.00* | CRA |
|  | Manon Coll. Anc | BEL | *50.83* | *4.00* | CRA |
|  | 456.21 | BEL | *50.83* | *4.00* | CRA |
|  | 456 GB X 514 | BEL | *50.83* | *4.00* | CRA |
| SP20 | Tresjuncos, Cuenca | ESP | 39.72 | -2.75 | IRTA |
| SP36 | Motril, Grenada | ESP | 36.75 | -3.52 | IRTA |
| SP44 | Zamarramala, Segovia | ESP | 40.97 | -4.13 | IRTA |
| SP111 | Coria del Rio, Sevilla | ESP | 37.29 | -6.05 | IRTA |
| SP114 | Sorzano, La Rioja | ESP | 42.33 | -2.53 | IRTA |
| SP131 | Allepuz, Teruel | ESP | 40.48 | -0.73 | IRTA |
| SP142 | Manacor, Mallorca | ESP | 39.57 | 3.20 | IRTA |
| SP143 | El Paso, Tenerife | ESP | 28.65 | -17.87 | IRTA |
| SP146 | Gutierre Monoz, Avila | ESP | 40.98 | -4.63 | IRTA |
| SP159 | Almazan, Soria | ESP | 41.48 | -2.53 | IRTA |
| 273 | Laari | FIN | 61.08 | 27.38 | NGB |
| 277 | Lähde | FIN | 60.97 | 23.02 | NGB |
| 308 | Veteläinen | FIN | 65.33 | 27.60 | NGB |
| 311 | Ruohonen | FIN | 65.00 | 27.02 | NGB |
| 314 | Rehakka-65 | FIN | 60.85 | 24.53 | NGB |
| 315 | Hakala | FIN | 60.65 | 27.82 | NGB |
| 317 | Kivistö | FIN | 60.93 | 24.07 | NGB |
| 320 | Haaraniemi | FIN | 62.90 | 29.38 | NGB |
| 326 | Rehakka-59 | FIN | 60.85 | 24.53 | NGB |
| 329 | Tiainen | FIN | 61.47 | 27.37 | NGB |
| 431 | Vesanto | FIN | 62.93 | 26.42 | NGB |
| 468 | Trysil | NOR | 61.32 | 12.27 | NGB |
| 1156 | Vanhala ME0402 | FIN | 61.47 | 27.37 | NGB |
| 2079 | Skjåk | NOR | 61.87 | 8.37 | NGB |
| 2107 | Refsum | NOR | 60.05 | 11.17 | NGB |
| 2565 | Kääs, Local Öland | SWE | 56.75 | 16.63 | NGB |
| 4413 | Ylenjoki AP0301 | FIN | 61.27 | 24.03 | NGB |
| 4431 | Pääskylä AP0201 | FIN | 61.42 | 23.52 | NGB |
| 4701 | Stjernebyg Fra Færøerne | FRO | 62.00 | -7.00 | NGB |
| 6927 | Uforædlet Jämtland | SWE | 63.43 | 14.07 | NGB |
| 9448 | Dønnes | NOR | 66.20 | 12.58 | NGB |
| 9511 | Langeland | DNK | 55.80 | 9.12 | NGB |
| 9529 | Lynderupgaard | DNK | 56.85 | 9.58 | NGB |
| HOR 63 | Heils Franken (Franconia) | DEU | *50.00* | *9.00* | IPK |
| HOR 64 | Kredlers Oberpfalzer (Pfalz) | DEU | *49.50* | *12.00* | IPK |
| HOR 66 | Strengs Franken (Franconia) | DEU | *50.10* | *9.10* | IPK |
| HOR 70 | Reiser Typ A (Jura) | DEU | *47.17* | *7.00* | IPK |
| HOR 76 | Firschers Wirchenbl (Lausitz) | DEU | *51.49* | *14.38* | IPK |
| HOR 82 | Hohenfinower (Oderbruch) | DEU | *50.32* | *14.22* | IPK |
| HOR 83 | Heines Vierzeilge (Brandenburg) | DEU | *53.00* | *14.00* | IPK |
| HOR 104 | Oldenburger (Oldenburg) | DEU | *53.04* | *8.36* | IPK |
| HOR 329 | Kneifels volkorngerste | DEU | *47.65* | *13.02* | IPK |
| HOR 354 | Schmids Messkircher Landgerste | DEU | *47.59* | *9.06* | IPK |
| HOR 357 | Dornberger Vierzeillige Futtergerste | DEU | *52.02* | *11.52* | IPK |
| HOR 1314 | Breisgauer Wintergerste Typ B | DEU | *47.59* | *7.51* | IPK |
| HOR 1610 | Dolhauer Kleingerste | DEU | *50.16* | *11.56* | IPK |
| HOR 2165 | Gorsdorfer D Gerste | DEU | *51.47* | *12.52* | IPK |
| HOR 17256 | Hiltegerste (Schwabia) | DEU | *48.33* | *10.50* | IPK |
| HOR 17358 | Neudorfer Marien (Neudorf) | DEU | *51.36* | *11.06* | IPK |
| HOR 19228 | Ettersberger (Schweilsig Holstein) | DEU | *54.00* | *10.50* | IPK |
| 1783 | Arkhangelsk | RUS | 64.57 | 40.53 | VAV |
| 1883 | Tambov | RUS | 52.73 | 41.43 | VAV |
| 1921 | Saratov | RUS | 51.57 | 46.03 | VAV |
| 1955 | Kaluga | RUS | 54.54 | 36.27 | VAV |
| 2018 | Leningrad | RUS | 60.00 | 32.00 | VAV |
| 2021 | Kursk | RUS | 51.73 | 36.19 | VAV |
| 2143 | Estonia | EST | 59.00 | 26.00 | VAV |
| 2156 | Karelia | RUS | 64.00 | 32.00 | VAV |
| 2544 | Estonia | EST | 59.10 | 26.10 | VAV |
| 2622 | Kherson | UKR | 46.63 | 32.60 | VAV |
| 3691 | Yaroslavl | RUS | 57.62 | 39.87 | VAV |
| 4120 | Vitsyebsk | BYS | 55.19 | 30.19 | VAV |
| 4121 | Vladimir | RUS | 56.14 | 40.40 | VAV |
| 4160 | Kaluga | RUS | 54.54 | 36.27 | VAV |
| 4185 | Perm | RUS | 58.00 | 56.25 | VAV |
| 4201 | Simbirsk | RUS | 54.33 | 48.40 | VAV |
| 4289 | Kirov | RUS | 58.60 | 49.66 | VAV |
| 4372 | Voronezh | RUS | 51.67 | 39.17 | VAV |
| 4379 | Ivanovo | RUS | 56.99 | 40.99 | VAV |
| 4429 | Volgograd | RUS | 48.80 | 44.59 | VAV |
| 4480 | Stavropol | RUS | 45.04 | 41.97 | VAV |
| 4522 | Vitsyebsk | BYS | 55.19 | 30.19 | VAV |
| 4535 | Orel | RUS | 52.97 | 36.08 | VAV |
| 4779 | Donetsk | RUS | 48.00 | 37.80 | VAV |
| 4788 | Dnipropetrovsk | RUS | 48.45 | 34.98 | VAV |
| 4965 | Omsk | RUS | 55.00 | 73.40 | VAV |
| 5314 | Voronezh | RUS | 51.67 | 39.17 | VAV |
| 6420 | Minsk | BYS | 53.90 | 27.57 | VAV |
| 10604 | Riga | LAT | 57.00 | 24.08 | VAV |
| 12006 | Poltava | UKR | 49.58 | 34.57 | VAV |
| 12216 | Crimea | UKR | 45.00 | 34.00 | VAV |
| 12828 | Zakarpatska | UKR | 48.33 | 23.00 | VAV |
| 13068 | Krasnodar | UKR | 45.03 | 38.98 | VAV |
| 14130 | Odesa | UKR | 46.47 | 30.73 | VAV |
| 17911 | Lithuania | LIT | 56.00 | 24.00 | VAV |
| 17914 | Lithuania | LIT | 56.00 | 24.00 | VAV |
| 18251 | Zakarpatska | UKR | 48.33 | 23.00 | VAV |
| 18317 | Kaliningrad | LAT | 54.75 | 21.50 | VAV |
| 18318 | Jelgava | LAT | 56.65 | 23.70 | VAV |
| 18342 | Ternopil | UKR | 49.55 | 25.58 | VAV |
| 18346 | Ternopil | UKR | 49.55 | 25.58 | VAV |
| 18593 | Satu-Mare (uyezd) | ROM | 47.80 | 22.88 | VAV |
| 18594 | Constanta (uyezd) | ROM | 44.25 | 28.33 | VAV |
| 18595 | Teleorman (uyezd) | ROM | 44.08 | 25.17 | VAV |
| 18596 | Ialomita (uyezd) | ROM | 44.67 | 27.00 | VAV |
| 18597 | Alba (uyezd) | ROM | 46.17 | 23.58 | VAV |
| 18598 | Arad (uyezd) | ROM | 46.18 | 21.32 | VAV |
| 18599 | Cluj (uyezd) | ROM | 46.77 | 23.60 | VAV |
| 3584 | Scotch Common Scottish Land Variety | GBR | *56.00* | *-4.00* | JIC |
| 7039 | Craigs Triumph Common Barley Selection | GBR | *56.00* | *-4.00* | JIC |
| 7049 | Rogue Rogue from Craigs Triumph Crop | GBR | *56.00* | *-4.00* | JIC |
| 7683 | Common Unknown | GBR | *56.00* | *-4.00* | JIC |
| 7046 | Tiree 6-Row Unknown | GBR | 56.50 | -6.92 | JIC |
| 4815 | Early Welsh Welsh Land Variety | GBR | *52.50* | *-3.50* | JIC |
| 24305 | Early Welsh Welsh Land Variety | GBR | *52.50* | *-3.50* | JIC |
| 7163 | Hen Gymro Welsh Land Variety | GBR | *52.50* | *-3.50* | JIC |
| 7164 | Hen Gymro Welsh Land Variety | GBR | *52.50* | *-3.50* | JIC |
| 7165 | Hen Gymro Welsh Land Variety | GBR | *52.50* | *-3.50* | JIC |
| 7166 | Hen Gymro Welsh Land Variety | GBR | *52.50* | *-3.50* | JIC |
| 7278 | Hen Gymro Welsh Land Variety | GBR | *52.50* | *-3.50* | JIC |
| 3482 | Hen Haidd Eulli Welsh Land Variety | GBR | *52.77* | *-4.80* | JIC |
| 4820 | St Davids Welsh Land Variety | GBR | *51.88* | *-5.27* | JIC |
| 24143 | Goldthorpe Chevalier Selection | GBR | *53.53* | *-1.30* | JIC |
| 4817 | Chevalier D10 English Variety Selection | GBR | *53.53* | *-1.30* | JIC |
| 7742 | Chevallier Land variety selection (Debenham, Suffolk by J. Chevalier) | GBR | *52.22* | *1.18* | JIC |
| 3585 | Swanneck English Land Race | GBR | *52.00* | *-1.00* | JIC |
| 3675 | Swannek Swanneck (English) Selection | ZAF | *52.00* | *-1.00* | JIC |
| 8204 | Swanneck Diamant Selection | GBR | *52.00* | *-1.00* | JIC |
| 4854 | Watts Watts Chatteris Single Plant Selection | GBR | *52.45* | *0.05* | JIC |
| 3561 | Prize Prolific English Variety Selection | GBR | *52.00* | *-1.00* | JIC |
| 3491 | Irish Goldthorpe English Variety Selection | IRL | *53.00* | *-8.00* | JIC |
| 4827 | Webbs Winter Unknown | GBR | *52.00* | *-1.00* | JIC |
| 24313 | Webbs Burton Malting Unknown | GBR | 52.80 | -1.62 | JIC |
| 3423 | Burtons Malting English Land Race | GBR | 52.80 | -1.62 | JIC |
| 8201 | Old Cornish Unknown | GBR | *50.42* | *-4.75* | JIC |
| 8200 | Long Eared Nottingham English Land Race | GBR | 52.97 | -1.17 | JIC |
| 3567 | Prior Chevalier Selection | AUS | *52.00* | *-1.00* | JIC |
| 7009 | Morayshire Gold Unknown | GBR | 57.42 | -3.25 | JIC |
| 24310 | Plumage Scandanavian Barley Selection | GBR | *62.00* | *15.00* | JIC |
| 7439 | Carters 4-Row Unknown | GBR | *52.00* | *-1.00* | JIC |
| 4834 | Plumage 63 Selection from Plumage | GBR | *62.00* | *15.00* | JIC |
| 7036 | Webbs Naked 2-Row Unknown | GBR | *52.00* | *-1.00* | JIC |
| 4835 | Beavans 35 Unknown | GBR | *52.00* | *-1.00* | JIC |
| 24308 | Millenium Unknown | GBR | *52.00* | *-1.00* | JIC |
| 4853 | Commercial English Land Race | GBR | *52.00* | *-1.00* | JIC |
| 3547 | Old Wiltshire English Land Race | GBR | 51.25 | -1.92 | JIC |
| 7455 | Winter (Seale Hayne) Unknown | GBR | 50.53 | -3.60 | JIC |
| 4831 | Carters 6-Row Unknown | GBR | *52.00* | *-1.00* | JIC |
| 3446 | Ducksbill English Land Race | GBR | *52.00* | *-1.00* | JIC |
| 3586 | Streatly English Land Race | GBR | 51.93 | -0.43 | JIC |
| 4845 | Golden Melon English Land Race | GBR | *52.00* | *-1.00* | JIC |
| 8149 | Padstow English Land Race | GBR | 50.53 | -4.93 | JIC |
| 9930 | Sarah Champagne Barley Selection | FRA | *49.00* | *4.50* | JIC |
| 4818 | Golden Drop English Variety Selection | GBR | *52.00* | *-1.00* | JIC |
| 4819 | Golden Pheasant English Variety Selection | GBR | *52.00* | *-1.00* | JIC |
| 4837 | Beavans 35/51 Unknown | GBR | *52.00* | *-1.00* | JIC |
| 7047 | Northumberland Rogue Unknown | GBR | *55.25* | *-2.00* | JIC |
| 3552 | Plumage Archer Plumage Archer | GBR | *52.00* | *-1.00* | JIC |
| 24145 | Plumage Archer Plumage Archer | GBR | *52.00* | *-1.00* | JIC |
| 7735 | Plumage Archer Plumage Archer | GBR | *52.00* | *-1.00* | JIC |
| 7027 | Plumage Archer Selection Plumage Archer Selection | GBR | *52.00* | *-1.00* | JIC |
| 4020 | Kenia Binder Gull | DNK | *56.00* | *10.00* | JIC |
| 7048 | Kenia Tall Unknown | DNK | *56.00* | *10.00* | JIC |
| 7551 | Fero Kenia Selection | DNK | *56.00* | *10.00* | JIC |
| 7457 | Archer Old Land Variety Selection | GBR | *52.00* | *-1.00* | JIC |
| 3425 | Beavans Archer English Land Race | GBR | *52.00* | *-1.00* | JIC |
| 8242 | Gartons Archer Unknown | GBR | *52.00* | *-1.00* | JIC |
| 3492 | Irish Archer English Variety Selection | IRL | *53.00* | *-8.00* | JIC |
| 4816 | Old Wiltshire Archer English Land Race | GBR | *51.25* | *-1.92* | JIC |
| 24144 | Old Wilts Archer Unknown | GBR | *51.25* | *-1.92* | JIC |
| 3385 | Archplume English Land Race | GBR | *52.00* | *-1.00* | JIC |
| 4836 | Gartons Winter Archer Unknown | GBR | *52.00* | *-1.00* | JIC |
| 7192 | Gotlands Unknown | SWE | *57.50* | *18.55* | JIC |
| 4824 | Groningen Dutch Land Variety | NLD | *53.22* | *6.55* | JIC |
| 9926 | Gloire du Velay Upper Loire Barley Selection | FRA | *45.33* | *3.67* | JIC |
| 7438 | Vindicat Groningen Land Race Selection | NLD | *53.22* | *6.55* | JIC |
| 7175 | Vollkorngerste Unknown | AUT | *47.33* | *13.33* | JIC |
| 7038 | Camton Spratt Archer*Goldthorpe | GBR | *52.00* | *-1.00* | JIC |
| 9931 | Standwell  Golden Melon*Fan | GBR | *52.00* | *-1.00* | JIC |
| 7698 | Hunter  Spratt Archer*Kenia | IRL | *53.00* | *-8.00* | JIC |
| 4840 | New Cross  Chevalier*Spratt Archer | GBR | *52.00* | *-1.00* | JIC |
| 3452 | Earl Spratt Archer Selection | GBR | *52.00* | *-1.00* | JIC |
| 8003 | Spratt Archer Irish Archer*Spratt | IRL | *53.00* | *-8.00* | JIC |
| 7051 | Bonus Maja*(Victory=Seger*Opal) | SWE | *62.00* | *15.00* | JIC |
| 8584 | Rene Guillemart Maja Selection | FRA | *56.00* | *10.00* | JIC |
| 7999 | Maja Binder*Gull | DNK | *56.00* | *10.00* | JIC |
| 8012 | Goldfield Golden Promise*Emir | GBR | *52.00* | *-1.00* | JIC |
| 24312 | Webbs Binder Unknown | GBR | *52.00* | *-1.00* | JIC |
| 24304 | D.K.S. Binder Unknown | GBR | *52.00* | *-1.00* | JIC |
| 4812 | Scotch Annat Scottish Land Variety | GBR | 56.70 | -2.43 | JIC |
| 24300 | Annat Unknown | GBR | 56.70 | -2.43 | JIC |
| 4822 | Old Cromarty Scottish Land Variety | GBR | 57.67 | -4.03 | JIC |
| 3962 | Bere Scottish Land Variety | GBR | *56.00* | *-4.00* | JIC |
| 4828 | Bere Scottish Land Variety | GBR | *56.00* | *-4.00* | JIC |
| 4843 | Bere Scottish Land Variety | GBR | *56.00* | *-4.00* | JIC |
| 20562 | Bere (Scots) Scottish Land Variety | GBR | *56.00* | *-4.00* | JIC |
| 7053 | Hen Gymro Welsh Land Variety | GBR | *52.50* | *-3.50* | JIC |
| 7054 | Hen Gymro Welsh Land Variety | GBR | *52.50* | *-3.50* | JIC |
| 7055 | Hen Gymro Welsh Land Variety | GBR | *52.50* | *-3.50* | JIC |
| 7056 | Hen Gymro Welsh Land Variety | GBR | *52.50* | *-3.50* | JIC |
| 7057 | Hen Gymro Welsh Land Variety | GBR | *52.50* | *-3.50* | JIC |
| 7058 | Hen Gymro Welsh Land Variety | GBR | *52.50* | *-3.50* | JIC |
| 7060 | Hen Gymro Welsh Land Variety | GBR | *52.50* | *-3.50* | JIC |
| 7893 | Aurore Kenia Selection | FRA | *46.00* | *2.00* | JIC |

Table S2. Microsatellite loci and PCR details

| Microsatellite | SCRI code | Primer sequences (5´→3´)^1^ | Chromosome |
| --- | --- | --- | --- |

SSR 3 SCSSR25691 ACGAGCTGATATCCCACGAG 3H

TCCGAGCTTCTTATCTTTGG

SSR 8 SCSSR04056 CCCATGAAGCCTCTTTACG 7H

GGAACGGAGGGAGTATTAAGC

SSR 9 SCSSR03907 CTCCCATCACACCATCTGTC 5H

GACATGGTTCCCTTCTTCTTC

SSR 10 SCSSR10477 AGAGCAATGAGCTCCTACCC 1H

GCTTACTCGCTCGTTTAGTCG

SSR 11 SCSSR14079 AAAATAAGGTTTCTTGTTCTTGG 4H

GAAACCCTGTTGAAGTACGG

SSR 12 SCSSR07970 TGCATTGGGAGTGCTAGG 7H

TGCAAGAAGCCAAGAATACC

SSR 13 SCSSR02748 GGTGCATTTGGAAGTCTAGG 1H

ATAGCAAGTGCCAAGTGAGC

SSR 14 SCSSR15864 GCATAAACGGGTGTAAGAGC 7H

CATCCAGTTCAGAGGATAGAGC

SSR 15 SCSSR02306 TGCCTTGTTTATGTAATATCTTGTG 5H

GGCGTAAATAAGAGTGTCTTCAG

SSR 16 SCSSR09398 AGAGCGCAAGTTACCAAGC 6H

GTGCACCTCAGCGAAAGG

SSR 17 SCSSR05939 TCATTGGGCTCTTCTACGG 5H

GCAAACCGGACTAAGTATGC

SSR 18 SCSSR18005 TCCTCACACAGAGAGAAGTGC 4H

CCCACACGGTGTAGTAGAGG

SSR 19 SCIND16991 CGCCGTTCCAGTTTAACTTC 5H

GGGCTTCCCCTCCTTTGTAT

SSR 20 SCSSR00103 GGTAAGGAGTGGGTCTCAGG 6H

CAAGCAGATGCAACTACACC

SSR 21 SCSSR07106 GCGCTGTCTCTTCTATGTGC 5H

AGGTGCTCCTAATCTGATGG

SSR 22 SCIND60002 CGAATGCAGTACAGCCTCAG 6H

CATCATCACGCCACCATACT

SSR 23 SCSSR08447 AAATTTGTATTGGCTGGTTCC 2H

ACAAAAGCAAACCCTAGACC

SSR 24 SCSSR10148 AAGCAGCAAAGCAAAGTACC 5H

TCATCAGCATCTGATCATCC

SSR 25 SCSSR07759 GCAACTCCTCATCATCTCAGG 2H

CAACAGCCAGAAGGTCTACG

SSR 26 SCSSR20569 ATCGAGCACCTACGAACC 3H

TTGCATAGCGGAAGTAATCC

SSR 27 SCSSR05599 TTCCATCATAACAGCAATGG 6H

TTCGTCGAAGGCTATGTAGG

SSR 28 SCSSR00334 CAAACAGCCACTGTCCTAGC 2H

AGGGCGAGGTAGATGACG

SSR 29 SCIND02587 GGTGACCCAGCCAAATTTTA 5H

GCAGCTGCTAGTTGGTTCATC

^1^Primer sequences kindly provided by J. Russell and L. Ramsay (Scottish Crop Research Institute)

Table S3. Microsatellite data for the 651 barley landraces

Microsatellite Number of Allele sizes Major allele Gene PIC Missing

alleles (bp) frequency frequency data %

SSR 3 7 218–236 0.80 0.33 0.30 12.4

SSR 8 26 132–198 0.20 0.90 0.89 7.1

SSR 9 25 113–175 0.17 0.91 0.90 12.9

SSR 10 22 131–189 0.50 0.71 0.70 36.6

SSR 11 5 140–151 0.83 0.29 0.28 2.9

SSR 12A 11 150–237 0.46 0.72 0.69 3.1

SSR 12B 11 243–291 0.42 0.74 0.71 11.2

SSR 13 4 151–158 0.46 0.60 0.52 6.0

SSR 14 7 170–189 0.87 0.24 0.22 16.3

SSR 15 5 151–159 0.57 0.58 0.51 1.1

SSR 16 13 163–201 0.46 0.69 0.65 2.5

SSR 17 6 164–183 0.90 0.19 0.18 3.8

SSR 18 3 183–187 0.95 0.09 0.09 3.7

SSR 19 6 167–184 0.75 0.37 0.33 24.3

SSR 20 5 168–176 0.32 0.73 0.68 1.7

SSR 21 7 171–186 0.45 0.62 0.54 4.9

SSR 22 8 177–221 0.74 0.42 0.39 41.9

SSR 23 4 181–187 0.45 0.67 0.61 7.7

SSR 24 10 181–216 0.59 0.57 0.51 3.5

SSR 25 12 181–228 0.38 0.69 0.64 0.8

SSR 26 6 185–196 0.61 0.57 0.53 19.7

SSR 27 6 183–198 0.49 0.63 0.56 2.3

SSR 28 5 203–209 0.97 0.05 0.05 2.3

SSR 29 2 204–205 0.86 0.24 0.21 1.4

Mean 9.0 0.59 0.53 0.49 9.6

Table S4. *PPD–H1* genotypes for 82 barley landraces

Genotype^1^ Accession source numbers

*Ppd–H1* 456.21, 3567, 3675, PI 183632, HOR 10366, HOR 10779, HOR 10943, HOR

11003, HOR 11113, HOR 11116, HOR 11118, HOR 3259, HOR 8814, HOR 8845,

HOR 991, Ligne 541

*ppd–H1* 3385, 3423, 3452, 3482, 3492, 3547, 3584, 3586, 4817, 4818, 4819, 4824, 4834,

4836, 4837, 7047, 7164, 7455, 7457, 8149, 9926, 9931, 24144, 24145, 24308,

24310, 24312, 24313, HOR 10630, HOR 7533, HOR 7535, HOR 8809, HOR

8810, HOR 8815, HOR 9277, HV0040, HV0041, HV0042, HV0063, HV0065,

HV0079, HV0080, HV0082, HV0084, HV0109, HV0110, HV0153, HV0242,

HV0245, HV0503, HV0556, HV0573, HV0574, HV0577, HV0583, HV0585,

HV0588, HV0595, HV0596, HV0597, HV0599, HV0645, HV0655, HV0696,

HV0699, HV0709

**^1^** Genotypes were assigned as daylength responsive (*Ppd–H1*), equivalent to early flowering, and daylight nonresponsive (*ppd–H1*), equivalent to late flowering, by typing the C/T polymorphism referred to as SNP48 (Jones *et al*., 2008)

Table S5. Geographical data

Population Number of Mean centre Mean centre Standard Mean pairwise

landraces longitude latitude distance (km) distance (km)

1 135 11.77 49.37 1278 1550

2 60 4.70 51.10 1513 1438

3 77 9.52 46.72 199 140

4 28 15.58 48.82 753 880

5 36 17.76 47.36 1668 2384

6 57 20.40 45.49 1184 1731

7 92 20.72 55.06 1679 2112

8 57 11.03 47.23 1274 1534

9 109 14.49 39.44 1163 1305

Figure S1. Expanded view of the core distribution of population 3. The locations of the individual landraces are indicated and the circle is the standard deviation ellipses for this population. All of the landraces in this region have a proportional population membership of ≥0.9.


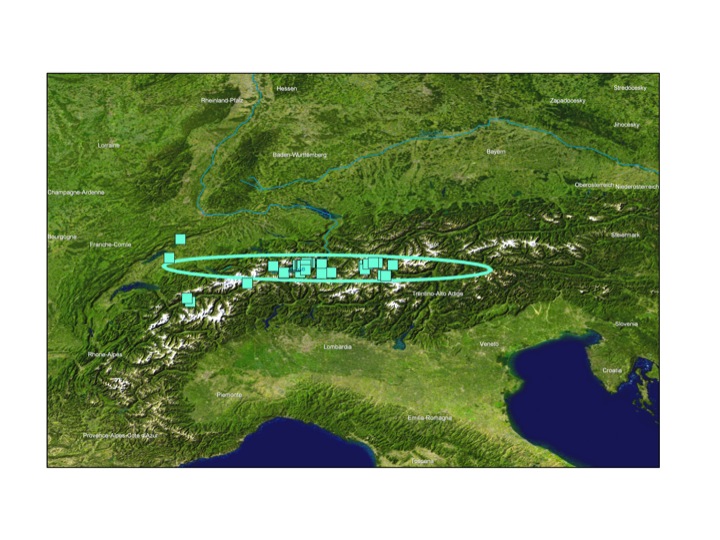

Supplement: Additional file 1 — Additional file for 'Evolutionary history of barley cultivation in Europe revealed by genetic analysis of extant landraces'. Contains Table S1 Barley accessions used in this study, Table S2 Microsatellite loci and PCR details, Table S3 Microsatellite data for the 651 barley landraces, Table S4 PPD-H1 genotypes for 82 barley landraces, Table S5 Geographical data, Figure S1 Expanded view of the core distribution of population 3. [file 1471-2148-11-320-S1.DOC]
